# Influence of camera geometry on 3D joint angle estimation in markerless motion capture

**DOI:** 10.3389/fspor.2026.1850018

**Published:** 2026-07-13

**Authors:** Xiong Zhao, Yan Zhang, Ryan B. Graham

**Affiliations:** 1 School of Human Kinetics, Faculty of Health Sciences, University of Ottawa, Ottawa, Ontario, Canada; 2 Google LLC, Mountain View, California, United States

**Keywords:** 3D kinematics, biomechanical analysis, camera geometry, markerless motion capture, pose estimation

## Abstract

**Objectives:**

This study examined how camera geometry influences the agreement of two-camera markerless joint-angle estimates with an eight-camera markerless reference system.

**Methods:**

Twenty-three female soccer athletes completed pre-season Y-balance, L-hop, countermovement jump, broad jump, and bodyweight squat tasks. Movements were recorded using an eight-camera Theia3D markerless system as the reference. All 28 two-camera combinations were processed using a custom Pose2Sim-based pipeline to estimate lower-limb joint angles. Camera geometry was quantified from calibration data, including camera height, distance to origin, azimuth, field of view, and horizontal angular separation. Agreement with the reference system and consistency among two-camera configurations were assessed using root mean square error (RMSE), mean absolute error (MAE), bias, limits of agreement, and coefficient of multiple correlation (CMC).

**Results:**

Agreement varied by camera geometry, anatomical plane, and task. Front and Back configurations, with moderate horizontal angular separation values of 68.59 ± 17.68° and 80.95 ± 22.35°, generally showed more favorable agreement, particularly for sagittal-plane hip and knee kinematics (RMSE < 8°, CMC ≈ 1.00). Same Quadrant configurations had the smallest angular separation (28.24 ± 3.81°) and poorer agreement, especially for frontal- and transverse-plane angles (RMSE > 10°, CMC < 0.60). Diagonal configurations had the largest angular separation (160.56 ± 13.40°) but did not consistently improve agreement. Y-balance and L-hop showed reduced agreement and higher dropout due to self-occlusion and participants moving outside the field of view.

**Conclusion:**

Camera geometry influenced two-camera markerless motion capture agreement. Front and Back configurations with moderate angular separation were most suitable for sagittal-dominant bilateral tasks, whereas Same Quadrant configurations should be avoided. For Y-balance and L-hop, camera placement should prioritize foot/ankle visibility and full movement-path coverage rather than angular separation alone.

## Introduction

Accurate estimation of three-dimensional (3D) joint kinematics from video-based motion capture depends fundamentally on camera geometry, including view separation, overlap, and perspective (1). In multi-view reconstruction, the spatial arrangement of cameras directly influences triangulation accuracy, depth resolution, and susceptibility to occlusion (2). While traditional marker-based systems achieve high accuracy through controlled camera configurations and calibrated volumes, emerging markerless motion capture approaches rely on computer vision algorithms that may be more sensitive to suboptimal camera placement (3).

Recent advances in markerless motion capture have enabled 3D kinematic estimation using multiple synchronized cameras without the need for reflective markers. Systems such as Theia3D (Theia Markerless Inc., Kingston, ON, Canada) and OpenCap have demonstrated promising validity relative to marker-based motion capture across a range of tasks (4–9). However, these approaches typically rely on predefined camera setups, and the influence of camera geometry on reconstruction accuracy remains incompletely understood, particularly for reduced camera configurations.

Two-camera markerless systems represent a practical and scalable alternative to multi-camera setups, but they introduce inherent geometric constraints. Limited angular separation between cameras can reduce depth resolution, while insufficient overlap may increase the likelihood of occlusion and tracking failure. These limitations are expected to disproportionately affect joint angle estimation in the frontal and transverse planes, where accurate 3D reconstruction requires robust multi-view information (3, 10).

Despite these challenges, few studies have systematically evaluated how different two-camera configurations influence 3D joint angle estimation across a range of dynamic movements. Most existing work has focused on validating specific camera setups rather than isolating the role of camera geometry as a determinant of reconstruction accuracy (7, 8, 11). A clearer understanding of these relationships is needed to guide both methodological development and practical implementation of markerless motion capture systems.

Therefore, the purpose of this study was to systematically evaluate the influence of camera geometry on 3D joint angle estimation using a two-camera markerless motion capture pipeline. Specifically, all pairwise combinations of an eight-camera system were assessed to determine how view separation and spatial configuration affect kinematic agreement across multiple movement tasks.

It was hypothesized that camera pairs with greater angular separation and overlapping fields of view would yield more accurate joint angle estimates, particularly in the sagittal plane, whereas configurations with limited separation would demonstrate reduced agreement due to decreased depth resolution and increased occlusion.

## Methods

### Participants

Twenty-five female varsity soccer players recruited from the University of Ottawa's women's soccer team but only twenty-three of them (mean age: 20.40 ± 1.98 years; height: 1.66 ± 0.06 m; mass: 63.31 ± 7.94 kg; BMI: 22.90 ± 2.30) participated the pre-season testing. Participants met the following inclusion criteria: (1)≥18 years of age; (2) able to understand English instructions; (3) fully cleared for athletic participation with no current musculoskeletal injury or concussion; and (4) able to complete 40 bilateral bodyweight squats. This study was approved by the Research Ethics Board at University of Ottawa (file #: H-07-24-10628). Participants were informed of their right to withdraw at any time without consequence, and all data were anonymized prior to analysis to ensure confidentiality.

### Protocol

**Figure 1:**
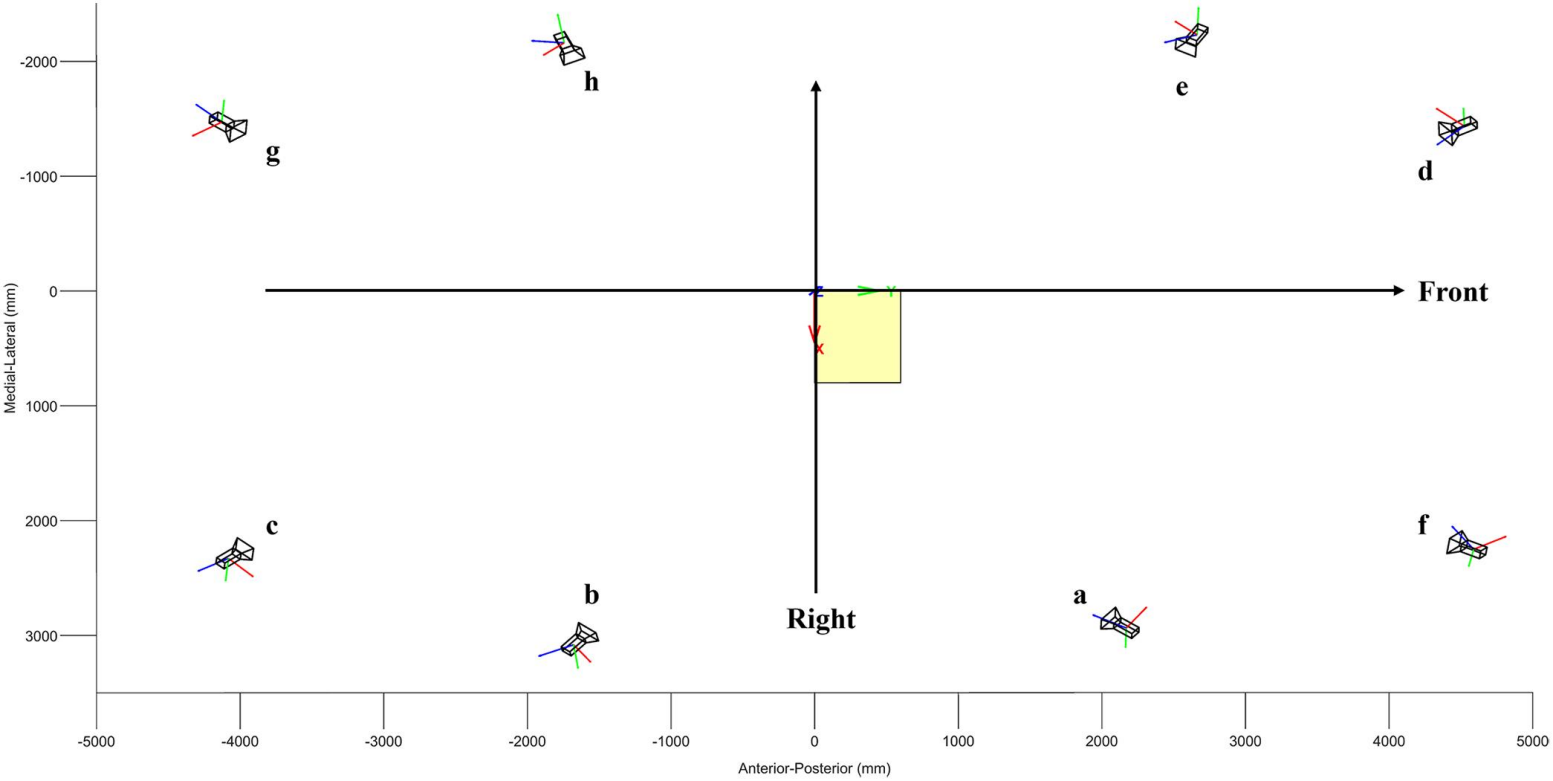
Bird's-eye view of the laboratory camera configuration. Eight cameras (a-h) are arranged around the capture volume to enable 3D tracking, with their individual coordinate systems shown. The global coordinate system is centered on the force plates (yellow rectangle), with the Y-axis aligned anterior–posterior, X-axis medial–lateral, and Z-axis vertical (mm). The capture volume is divided into four quadrants relative to the participant's orientation and the world coordinate system. “Front” indicates the direction faced by the participant during task execution, while “Right” refers to the participant's right-hand side..

Upon arriving at the laboratory, participants read and signed the informed consent form, and had their leg length, height, and weight measured. Once familiar with the lab and movements, they performed a single trial of T-balance with eyes open and Y-balance bilaterally, one trial of L-hop bilaterally, three repetitions of single-leg and bilateral countermovement jumps, and three broad jumps in a randomized order. After a minimum 5 min rest, participants completed 35 repetitions of bodyweight squats at 40 beats per minute (BPM) to a metronome. Whole-body kinematics were recorded using an eight-camera system consisting of Vicon Vue video cameras (Vicon Motion Systems Ltd., Oxford, UK; 1,920 × 1,080 pixels, 60 Hz), with markerless motion capture analysis performed using Theia3D (Theia Markerless Inc., Kingston, ON, Canada). The camera configuration, illustrated in Figure 1, remained consistent across all testing sessions throughout the season. However, the system was recalibrated prior to each data collection session to ensure measurement accuracy. During the same data collection session, participants also completed two reaction time tasks. These assessments were part of a broader testing battery but are beyond the scope of the present analysis. The full testing protocol, excluding the consent form and questionnaire, was repeated at mid- and post-season.

### Data processing

For validation purposes, only seven pre-season tasks: a single trial of Y-balance (YB) bilaterally, one trial of L-hop bilaterally, one repetition of bilateral countermovement jumps (CMJ), one broad jump (BJ). For the squat task, three repetitions were selected from each 35-repetition trial. The first five repetitions were discarded to minimize potential task-acclimation effects, and repetitions 6–8 were retained for analysis. Squat repetitions were segmented based on the vertical center-of-mass trajectory.

#### Theia3D data

Videos from all eight camera views were transferred and analyzed using Theia 3D Apollo v2024 (Theia Markerless Inc., Kingston, ON, Canada) with the default 17-segment model configuration and default GCVSPL smoothing cutoff frequency of 20 Hz. Three-dimensional joint kinematics were then processed in Visual3D (C-Motion Inc., Germantown, MD, USA) for each movement task. Final joint kinematics were exported to MATLAB R2019b (The MathWorks Inc., Natick, MA, USA), where they were low-pass filtered using a fourth-order zero-lag Butterworth filter with a 6 Hz cutoff frequency. These processed joint angles served as the reference for assessing agreement with the two-camera combinations.

#### Two-camera data

**Figure 2:**
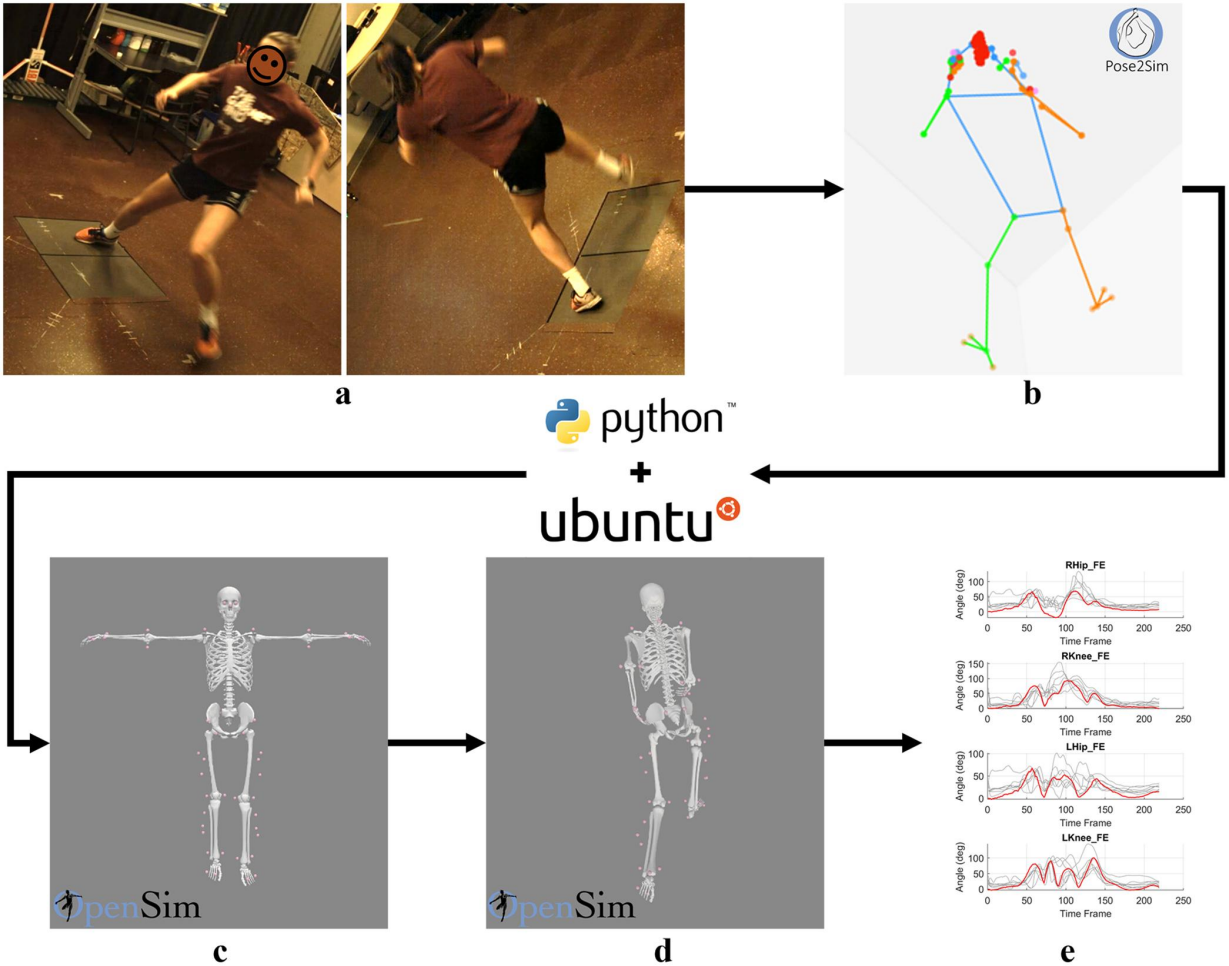
Overview of the workflow. (**a**) 2D image frames from two cameras during a movement task; (**b**) 3D pose estimation using the Pose2Sim pipeline with RTMPose; (**c**) scaling of a musculoskeletal model in OpenSim based on the estimated 3D pose; (**d**, **e**) computation of joint angles via inverse kinematics.

For two-camera motion capture pipeline, videos from every two of the eight camera views $\left(C_{8}^{2}=28\;combinations\right)$ were processed independently using a custom Python 3.10 script on Ubuntu 22.04 LTS. Each combination was treated as an individual input into a Pose2Sim (12) workflow in Figure 2.

Within the workflow, first, camera calibration was performed for each two-camera configuration using the same calibration file used in Theia3D, with only the two relevant cameras retained per combination. Following calibration, human pose estimation was conducted using RTMPose (13), a 2D keypoint detector. The detected keypoints were triangulated to obtain 3D coordinates using methods implemented in the Pose2Sim framework. To enhance the spatial resolution of the triangulated data, a pretrained Long Short-Term Memory (LSTM) model was applied to predict the positions of 52 anatomically relevant markers from the original 27 sparse keypoints (14). The resulting 3D coordinates were then low-pass filtered using a fourth-order zero-lag Butterworth filter with a 6 Hz cutoff frequency. Filtered marker trajectories were saved in OpenSim-compatible*.trc* format and imported into OpenSim 4.5 (15). Two-camera trajectories were processed in OpenSim using the Pose2Sim-provided *Model_Pose2Sim_Halpe26.osim* model configured for the Halpe26 keypoint set. In this model, the ankle is represented as a CustomJoint with one ankle flexion coordinate, and the subtalar joint is represented separately as a PinJoint, whereas Theia3D reports ankle rotations using three orthogonal Cartesian axes. The model was scaled, and inverse kinematics was performed to estimate lower-limb joint angles (12).

**Figure 3:**
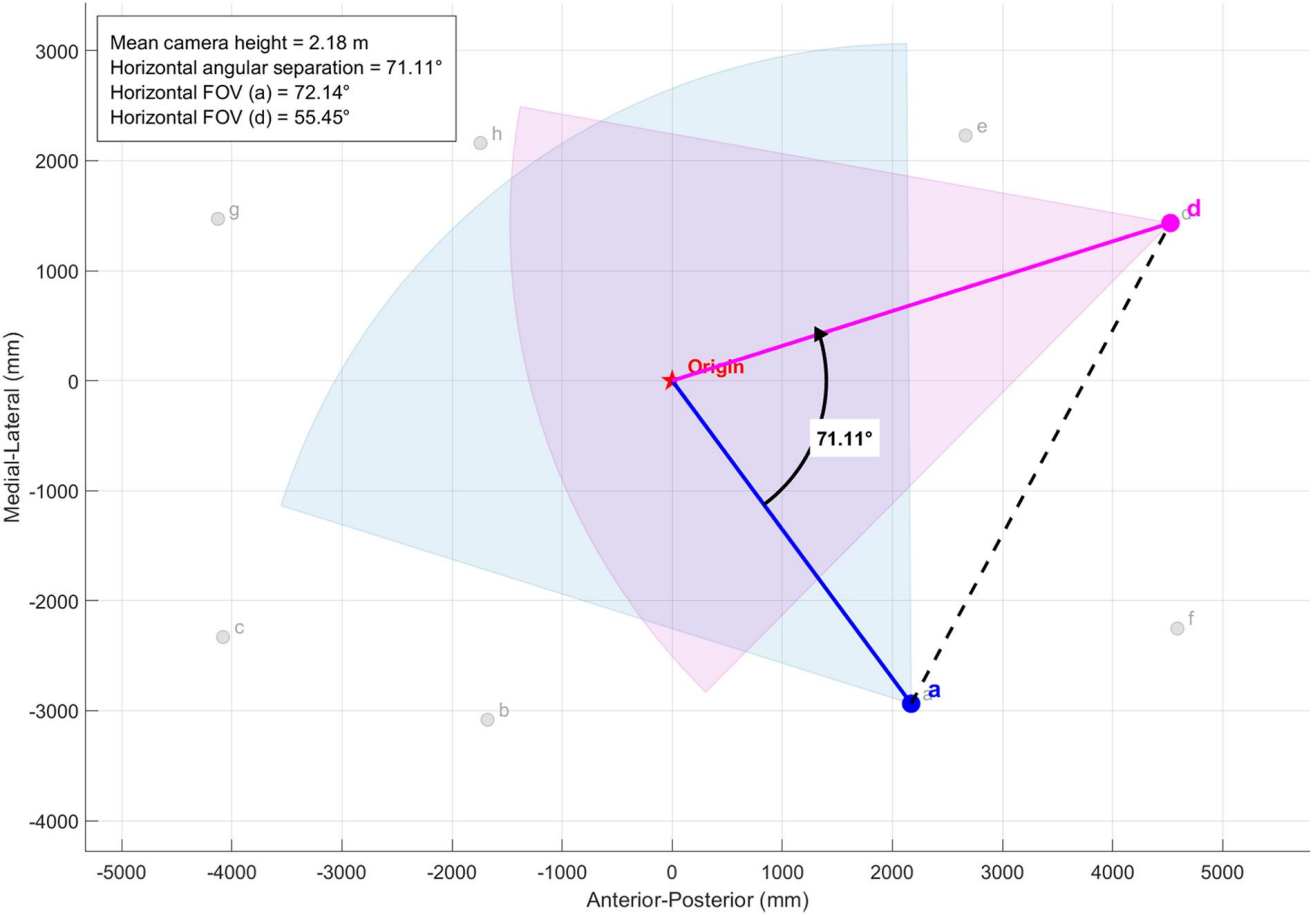
Representative example of one of the 28 two-camera configurations, shown for pair a_d (Front group). Mean camera height was 2.18 m, horizontal angular separation was 71.11°, and horizontal FOV was 72.14° for camera **a** and 55.45° for camera **d**. Solid lines indicate camera-center vectors, the dashed line indicates baseline distance, the black arc indicates angular separation, and shaded sectors indicate estimated horizontal FOV.

Camera intrinsic parameters (focal length and principal point) and extrinsic parameters (camera height, distance to the laboratory origin and azimuth) were extracted from the calibration file to characterize each two-camera configuration. For each camera, horizontal distance to origin was calculated from the X and Y coordinates, while three-dimensional distance to origin was calculated using the full X, Y, and Z coordinates. Horizontal angular separation (Figure 3) was calculated for each camera pair as the smaller angular difference between the azimuths of the two camera centers. Camera pairs were grouped into six geometry subgroups: Front, Back, Left, Right, Same Quadrant, and Diagonal (Supplementary Material 1, Table S1).

Pair-specific geometry visualizations for all 28 combinations are provided in Supplementary Material 1, Figures S1–S28. Horizontal and vertical fields of view (FOV) were estimated from calibrated focal length and image resolution (Supplementary Material 1, Tables S1, S2).

From both systems, joint angles from the following degrees of freedom were extracted for both left and right limbs for comparison: hip (flexion/extension, add-/ab-duction, internal/external rotation), knee (flexion/extension) and ankle (plantar-/dorsi-flexion, inversion/eversion). For each task, joint angle data from both Theia3D (reference) and each of the 28 two-camera combinations were trimmed from beginning and end of the task based on the same criteria and time-normalized to 100 frames to enable temporal alignment and error computation. Trials with unsuccessful pose estimation or triangulation, and incomplete tasks tracking from the two-camera combinations were excluded.

### Statistical analysis

#### Theia3D vs. two-camera combinations

Root mean square error (RMSE) and mean absolute error (MAE) were calculated for each individual trial, median RMSE and median MAE were calculated to quantify the magnitude of pointwise differences across the time-normalized joint angle trajectories. RMSE was treated as the primary metric because it penalizes larger deviations and is widely reported in markerless motion-capture validation studies. MAE was retained in the Supplementary Tables as a secondary metric representing typical absolute deviation with less sensitivity to isolated large errors. RMSE values were interpreted relative to the ∼5° threshold commonly used in three-dimensional gait-analysis reliability literature, with values below this threshold considered acceptable and values above this threshold interpreted cautiously as potentially meaningful measurement error (16). Median coefficient of multiple correlation (CMC) was calculated to assess waveform similarity across the movement cycle, as it captures both temporal alignment and amplitude patterns. Bland-Altman analyses were also performed using paired time-normalized joint-angle values to assess systematic bias and define limits of agreement (LoA) between systems. Each of these metrics was computed between each two-camera combination and the Theia3D baseline.

#### Within two-camera combinations

Median RMSE and CMC values were extracted for each joint and task. Since no gold-standard reference was available, and the number of combinations varied across groups, pairwise comparisons were conducted rather than comparisons to a group mean. Specifically, all possible pairwise differences in RMSE were computed within each group to characterize the internal consistency of joint estimates. The median and 25th–75th interquartile range (IQR) of these pairwise RMSEs and CMCs were used to summarize the agreement and correlation. This approach allowed for a more direct evaluation of variability within each group, independent of group size or any central tendency that may be skewed by outliers.

To interpret the strength of waveform agreement, CMC values were categorized according to established guidelines (17): values below 0.10 were considered negligible, 0.10–0.39 as weak, 0.40–0.69 as moderate, 0.70–0.89 as strong, and values from 0.90–1.00 as very strong. In parallel, RMSE and MAE were used as measures of prediction agreement, with lower values indicating greater proximity between the joint angle outputs of the two-camera combinations and those of the reference system (4, 6, 18).

Camera-geometry subgroup differences in focal length, camera height, distance -to-origin, FOV, and angular separation, were examined using one-way ANOVA, with Kruskal–Wallis tests as sensitivity analyses. All analyses were conducted in MATLAB R2019b (The MathWorks Inc., Natick, MA, USA).

## Results

Of the 4,508 potential trials, 3,999 (88.7%) were retained and 509 (11.3%) were excluded due to failed or incomplete reconstruction. Dropout rates were highest during Y-balance and L-hop trials (Table 1). Manual verification indicated that Y-balance failures were mainly related to limb crossing/self-occlusion, whereas L-hop failures were primarily caused by participants hopping outside the field of view of one or both cameras. Trial retention rate was therefore interpreted as a robustness metric in addition to kinematic agreement. For convenience, the term “between systems” refers to comparisons between the Theia3D system and each individual two-camera combination, while “within combinations” refers to comparisons among different two-camera configurations.

**Table 1:** Summary of the dropout rates by camera group and task for 2-camera combinations.

Group	Task				
	BJ	L-hop	YB	Squat	CMJ
Front	2.48%	1.09%	7.61%	0.93%	0.00%
Back	1.09%	4.97%	5.43%	0.00%	0.00%
Left	0.93%	6.21%	4.04%	0.78%	0.16%
Right	1.40%	0.78%	6.68%	0.93%	0.31%
Diagonal	1.09%	2.80%	6.99%	1.32%	0.08%
Same quadrant	1.24%	1.09%	5.59%	0.78%	0.00%

Front/Back: both cameras positioned anteriorly/ posteriorly (left and right); Left/Right: both cameras on the left/right side (front-left/right and back-left/right); Diagonal: two cameras placed diagonally; Same quadrant: both cameras positioned in the same quadrant (e.g., both front-left). BJ, broad jump; YB, Y-balance; CMJ, countermovement jump.

Across the eight cameras, the estimated horizontal and vertical FOV were 66.31 ± 8.30° and 40.50 ± 5.84°, respectively. Mean camera height was 2.39 ± 0.36 m, with a mean 3D distance-to-origin of 4.71 ± 0.77 m and a mean horizontal distance-to-origin distance of 4.04 ± 0.81 m. When summarized by camera-geometry subgroup, Front configurations (*a_e, a_d, e_f, d_f*) had a mean horizontal angular separation of 68.59 ± 17.68°, mean height of 2.41 ± 0.21 m, and mean horizontal FOV of 62.34 ± 4.56°. Back configurations (*b_h, b_g, c_h, c_g*) had a mean angular separation of 80.94 ± 22.35°, mean height of 2.37 ± 0.19 m, and mean horizontal FOV of 70.28 ± 3.88°. Left (*d_h, d_g, e_h, e_g*) and Right (*b_f, c_f, a_b, a_c*) configurations had angular separations of 115.88 ± 19.31° and 94.58 ± 20.95°, respectively, with mean heights of 2.57 ± 0.01 m and 2.21 ± 0.28 m. Same Quadrant configurations (*a_f, d_e, b_c, g_h*) had the smallest angular separation (28.23 ± 3.81°), while Diagonal configurations (*c_d, c_e, b_d, b_e, a_g, a_h, f_g, f_h*) had the largest angular separation (160.56 ± 13.40°). Detailed camera calibration parameters and subgroup definitions are provided in Supplementary Material 1, Tables S1, S2.

Across camera-geometry subgroups, focal length, horizontal/vertical FOV, camera height, and distance-to-origin were broadly comparable. In contrast, horizontal angular separation and baseline distance differed substantially by subgroup (Supplementary Material 1, Table S3).

### Between systems

#### By joint

When averaging across participants, tasks, and combinations, joint angle estimation performance varies depending on the anatomical plane of motion and the combination. In general, joint angles in the sagittal plane, including flexion/extension at the hip and knee, as well as ankle plantarflexion/dorsiflexion, demonstrated the lowest RMSE, and Bias values. However, hip flexion/extension consistently showed a substantial constant offset across all tasks, with RMSE ranging from 17.16° to 27.51°, and Bias from 10.23° to 26.71°, despite showing high waveform similarity with CMC values exceeding 0.85.

**Figure 4:**
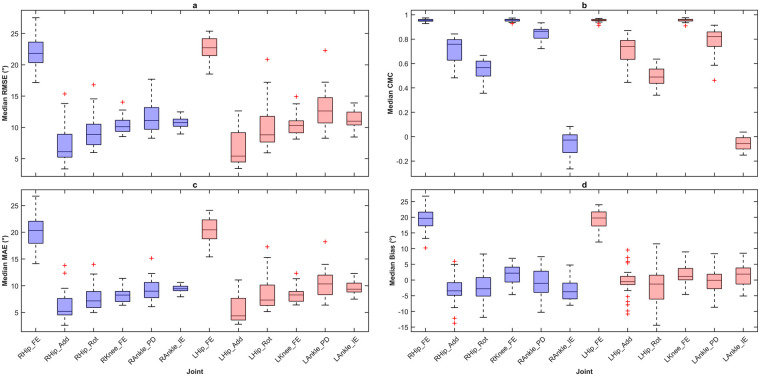
Boxplots of the four validation metrics comparing Theia3D and 2-camera markerless estimates across joints. **(a)** Root mean square error (RMSE), **(b)** coefficient of multiple correlation (CMC), **(c)** mean absolute error (MAE), and **(d)** bias. Values represent median metrics across all tasks and participants. Blue boxes indicate the right limb, and red boxes indicate the left limb. Red “+” symbols represent outliers.

In contrast, joint angles in the frontal and transverse planes (e.g., hip abduction/adduction, hip internal/external rotation, and ankle inversion/eversion) exhibited lower RMSE, and Bias, yet showed only moderate waveform agreement, with CMC values typically around 0.60. The poorest performance in terms of waveform similarity was observed for ankle inversion/eversion, where CMC values approached zero, indicating minimal alignment with the reference signal. These patterns are visualized in Figure 4, highlighting both joint-specific trends in agreement.

When agreement metrics were summarized by camera-geometry subgroup, Front and Back configurations generally showed the most favorable agreement with the Theia3D reference system, particularly for sagittal-plane joint angles (Table 2). For example, right hip flexion/extension RMSE was 4.20° in the Front group and 3.87° in the Back group, with CMC values ≥ 0.99. Similar patterns were observed for knee flexion/extension. In contrast, Same Quadrant configurations showed the least favorable agreement, particularly for frontal- and transverse-plane angles. In this subgroup, left hip rotation RMSE reached 15.77° with a CMC of 0.43, while left ankle inversion/eversion RMSE reached 14.76° with a CMC of 0.33. Diagonal configurations generally performed better than Same Quadrant configurations but showed higher errors and lower waveform similarity than Front and Back configurations for several non-sagittal joint angles. Left and Right configurations showed variable agreement, with favorable sagittal-plane performance but less consistent agreement for frontal- and transverse-plane motions. In contrast, the *a_f* combination (Same quadrant) was among the weakest performers. It consistently produced higher errors and weaker agreement across multiple joints. For instance, hip abduction/adduction showed an RMSE of 8.86° (6.79–12.11), and a low CMC of 0.48 (0.11–0.73). For ankle inversion/eversion, RMSE reached 13.83° (11.81–16.86), with CMC values close to zero, indicating poor tracking fidelity. Notably, hip rotation under *a_f* also showed elevated errors (RMSE = 11.70°; CMC = 0.50), with a systematic underestimation bias of −8.48° (−11.43 to −4.18).

**Table 2:** Camera-subgroup agreement metrics averaged across tasks and joints.

Group	Metrics	Hip_FE	Hip_Add	Hip_Rot	Knee_FE	Ankle_PD	Ankle_IE
		R/L	R/L	R/L	R/L	R/L	R/L
Front	RMSE	**4.20** (3.21, 6.08)/**4.07** (2.63, 6.77)	**3.51** (2.43, 5.00)/**3.54** (2.40, 5.06)	**5.37** (3.59, 7.89)/**6.04** (4.36, 8.24)	**3.59** (2.51, 5.65)/**4.47** (3.01, 6.77)	**5.95** (3.65, 10.6)/**6.68** (4.55, 11.5)	**4.98** (3.37, 7.30)/**5.48** (3.79, 7.73)
	CMC	**0.99** (0.97, 1.00)/**0.99** (0.97, 1.00)	**0.89** (0.69, 0.96)/**0.84** (0.60, 0.96)	**0.87** (0.65, 0.96)/**0.86** (0.66, 0.95)	**1.00** (0.98, 1.00)/**1.00** (0.98, 1.00)	**0.95** (0.86, 0.99)/**0.95** (0.83, 0.99)	**0.83** (0.59, 0.93)/**0.82** (0.61, 0.91)
Back	RMSE	**3.87** (2.93, 5.31)/**3.40** (2.60, 4.51)	**3.07** (2.17, 4.74)/**3.42** (2.52, 4.76)	**4.72** (3.13, 6.75)/**5.34** (3.70, 7.08)	**3.68** (2.72, 5.12)/**3.66** (2.77, 5.18)	**7.00** (4.88, 10.5)/**8.63** (6.18, 11.73)	**5.51** (3.19, 8.64)/**7.12** (4.93, 10.22)
	CMC	**1.00** (0.99, 1.00)/**1.00** (0.99, 1.00)	**0.86** (0.65, 0.95)/**0.85** (0.57, 0.95)	**0.91** (0.76, 0.97)/**0.82** (0.61, 0.92)	**1.00** (0.99, 1.00)/**1.00** (0.99, 1.00)	**0.93** (0.82, 0.97)/**0.85** (0.67, 0.93)	**0.68** (0.40, 0.85)/**0.65** (0.30, 0.84)
Left	RMSE	**6.34** (4.14, 9.31)/**4.61** (3.00, 7.47)	**4.90** (3.40, 7.21)/**4.43** (3.07, 6.53)	**6.07** (4.31, 8.73)/**7.23** (5.09, 9.86)	**5.13** (3.36, 8.70)/**4.82** (3.00, 9.95)	**8.13** (4.92, 13.7)/**6.83** (3.80, 12.22)	**6.38** (4.61, 8.99)/**5.92** (3.93, 8.91)
	CMC	**1.00** (0.97, 1.00)/**1.00** (0.97, 1.00)	**0.80** (0.49, 0.91)/**0.83** (0.62, 0.94)	**0.87** (0.69, 0.95)/**0.79** (0.52, 0.92)	**0.99** (0.97, 1.00)/**0.99** (0.96, 1.00)	**0.94** (0.81, 0.98)/**0.96** (0.83, 0.99)	**0.60** (0.29, 0.80)/**0.82** (0.54, 0.92)
Right	RMSE	**4.13** (2.89, 5.64)/**5.51** (3.81, 7.59)	**4.43** (3.26, 5.71)/**4.49** (3.40, 6.30)	**5.56** (3.76, 8.09)/**6.48** (4.47, 9.12)	**4.22** (2.96, 6.02)/**5.75** (4.20, 8.06)	**5.31** (3.33, 8.28)/**10.66** (6.68, 16.15)	**4.93** (3.57, 7.63)/**7.09** (4.94, 9.92)
	CMC	**1.00** (0.98, 1.00)/**0.99** (0.98, 1.00)	**0.93** (0.77, 0.97)/**0.83** (0.51, 0.96)	**0.90** (0.71, 0.96)/**0.82** (0.58, 0.91)	**1.00** (0.99, 1.00)/**0.96** (0.97, 1.00)	**0.96** (0.88, 0.98)/**0.86** (0.67, 0.94)	**0.86** (0.68, 0.94)/**0.64** (0.33, 0.85)
	RMSE	**8.74** (6.14, 12.0)/**8.10** (5.63, 11.1)	**12.52** (5.69, 19.46)/**13.09** (6.02, 20.14)	**14.13** (7.00, 20.59)/**17.06** (8.42, 24.66)	**7.20** (4.91, 10.0)/**7.34** (4.93, 10.45)	**10.41** (6.85, 15.37)/**11.59** (7.29, 16.88)	**7.87** (5.36, 11.10)/**9.86** (7.03, 13.00)
	CMC	**0.99** (0.95, 1.00)/**0.99** (0.96, 1.00)	**0.71** (0.39, 0.93)/**0.66** (0.40, 0.92)	**0.67** (0.32, 0.87)/**0.61** (0.18, 0.86)	**0.99** (0.97, 1.00)/**0.99** (0.96, 1.00)	**0.91** (0.73, 0.97)/**0.86** (0.66, 0.95)	**0.67** (0.34, 0.85)/**0.61** (0.23, 0.82)
Same quadrant	RMSE	**11.72** (8.93, 15.41)/**10.60** (7.32, 15.15)	**10.24** (7.78, 13.77)/**11.46** (8.66, 14.57)	**13.04** (9.46, 17.46)/**15.77** (11.05, 20.45)	**11.42** (7.90, 15.34)/**12.66** (9.20, 18.09)	**19.91** (13.36, 27.47)/**21.60** (14.35, 29.14)	**13.71** (9.51, 17.36)/**14.76** (10.88, 17.63)
	CMC	**0.96** (0.86, 0.99)/**0.96** (0.88, 0.99)	**0.42** (0.09, 0.77)/**0.28** (0.17, 0.66)	**0.52**(0.19, 0.77)/**0.43**(0.07, 0.68)	**0.96**(0.89, 0.99)/**0.95**(0.84, 0.99)	**0.70**(0.34, 0.87)/**0.48**(0.19, 0.77)	**0.30**(0.01, 0.59)/**0.33**(0.02, 0.60)

Values are reported as median (interquartile range), with median values shown in bold. RMSE is reported in degrees, and CMC represents waveform similarity. Results are summarized by camera-geometry subgroup and averaged across tasks and joints, with right/left limb values reported within each joint angle column. Lower RMSE and higher CMC indicate more favorable agreement with the eight-camera Theia3D reference system. Camera subgroup definitions are provided in Supplementary Table S1.

**Figure 5:**
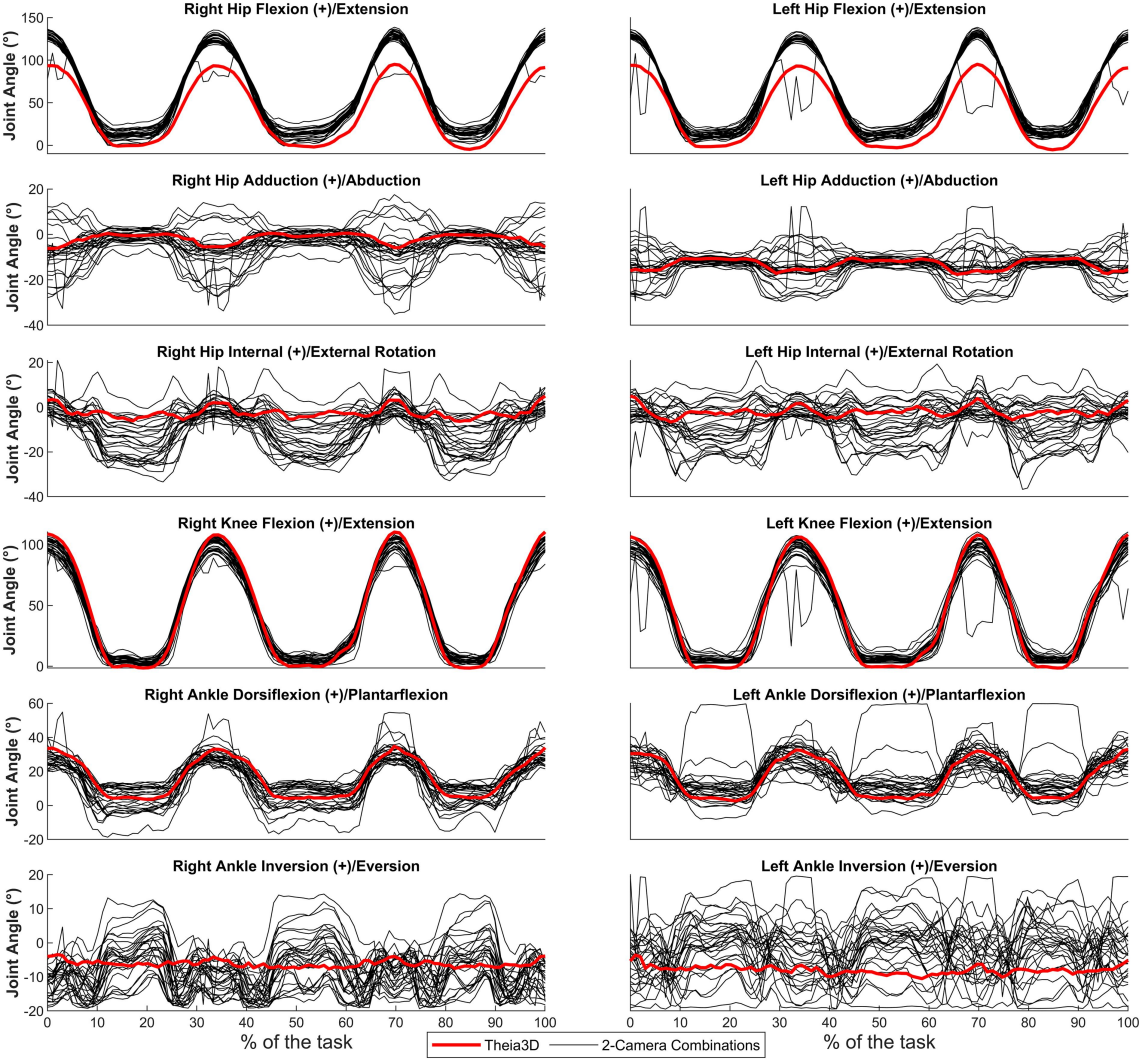
Example of lower limb joint angles during the squat task, comparing the Theia3D baseline (red) with outputs from all 2-camera combinations (black). Each black line represents the joint angle trajectory from a different camera combination. The x-axis denotes the percentage of the task cycle, and the y-axis indicates joint angle in degrees.

For example, the overall tracking performance is illustrated by the joint angle waveforms during the squat task shown in Figure 5. Complete joint-level agreement metrics across all camera combinations are provided in Supplementary Table S1.

#### By task

When examining the agreement between systems across tasks, notable variations in performance emerged depending on the task and the camera combination. Overall, camera combinations such as *d_f* (Front), *b_h* (Back), and *a_b* (Right) consistently produced joint kinematics across multiple tasks (Squat, BJ and CMJ) with relatively low RMSE, high waveform similarity, and minimal systematic bias. In contrast, combinations like *a_f* (Same quadrant), *c_d* (Diagonal) performed poorly in most tasks, particularly in unilateral tasks (L-hop and YB) where waveform agreement was reduced and bias was more pronounced.

Among the best-performing combinations, *b_h* (Back) and *a_b* (Right) showed strong agreement in Squat, BJ, and CMJ tasks, with consistently high CMC values and low errors across metrics (Supplementary Figures S1–S4). Combinations such as *a_c* (Right) and *d_f* (Front) also yielded favorable results in these tasks and performed moderately well in L-hop. Additionally, *c_h* and *b_g* from group Back provided adequate tracking in symmetric tasks, though performance varied more by joint. These combinations were effective for lower limb sagittal-plane movements but lacked consistency in frontal and transverse planes.

In contrast, combinations such as *a_f and h_g* (Same quadrant), and *c_d* (Diagonal) exhibited reduced performance across most tasks, especially in Y-balance and L-hop, where waveform agreement (CMC) was often poor and RMSE exceeded 15°. These diagonal and same-quadrant combinations frequently resulted in elevated bias values, as visualized in Supplementary Figure S4.

Among the tasks, Squat and BJ were generally captured more reliably across most camera combinations, indicated by higher overall CMCs and lower RMSEs. In contrast, tasks involving unilateral movements, such as Y-balance and L-hop, exhibited greater variability in performance depending on the camera combination. For instance, few configurations consistently performed well across all four metrics in left YB or right L-hop. Detailed task- and camera-combination-specific agreement metrics, including RMSE, waveform similarity, and bias, are provided in Supplementary Table S2.

### Within combinations

#### By joint

When analyzing agreement within two-camera combinations, performance by joint, task, and subgroup was evaluated. Across all joints and combinations (Table 3), flexion/extension in the sagittal plane, particularly at the hip and knee, showed the highest agreement. Median RMSE values for right and left hip flexion/extension were 7.71° (5.23–11.25°) and 7.39° (5.01–10.81°), respectively, with waveform similarity (CMC = 0.99). Knee flexion/extension followed a similar trend with RMSEs of 7.37° and 7.94° and CMCs of 0.99 for both limbs. In contrast, greater variability and lower CMCs were found in the frontal and transverse planes. Hip abduction/adduction and ankle inversion/eversion showed RMSEs in the 8–10° range and moderate to weak CMCs (0.53–0.71), especially in the left limb.

**Table 3:** Summary of joint angle median RMSE and CMC values averaged across all 2-camera combinations.

Joint	Median RMSE (IQR)		Median CMC (IQR)	
	Right	Left	Right	Left
Hip_FE	**7.71** (5.23, 11.25)	**7.39** (5.01, 10.81)	**0.99** (0.95, 1.00)	**0.99** (0.95, 1.00)
Hip_Add	**7.98** (5.05, 11.71)	**8.28** (5.21, 12.17)	**0.71** (0.26, 0.91)	**0.65** (0.18, 0.88)
Hip_Rot	**9.46** (6.10, 14.06)	**10.75** (6.97, 15.96)	**0.74** (0.45, 0.90)	**0.67** (0.34, 0.85)
Knee_FE	**7.37** (4.97, 10.72)	**7.94** (5.34, 11.64)	**0.99** (0.96, 1.00)	**0.99** (0.95, 1.00)
Ankle_PD	**11.27** (7.49, 17.04)	**12.92** (8.54, 19.35)	**0.88** (0.69, 0.96)	**0.81** (0.56, 0.94)
Ankle_IE	**8.88** (6.03, 12.42)	**10.05** (7.20, 13.58)	**0.56** (0.22, 0.80)	**0.53** (0.20, 0.77)

Values represent the median RMSE (in degrees) and CMC with interquartile ranges (IQR) for each joint angle, averaged across all 2-camera combinations and tasks. Results are reported separately for the right and left limbs. Higher CMC and lower RMSE values indicate better agreement.

Values are reported as median (interquartile range), with median values shown in bold.

#### By task

When agreement metrics were averaged across camera combinations by task, squats generally showed the lowest RMSE and highest waveform similarity across most joints (Supplementary Table S3), squat consistently yielded the highest agreement in joint angle estimations, with lower RMSEs and very high waveform agreement across most joints. Specifically, right and left hip flexion/extension demonstrated RMSEs of 5.44° and 5.40°, respectively, with CMCs of 1.00. Right and left knee flexion/extension followed closely with RMSEs of 6.19° and 6.11°, and CMCs also reaching 1.00. Ankle plantarflexion/dorsiflexion on both limbs showed RMSEs of 6.85° (right) and 6.25° (left), with CMCs ranging from 0.96 to 0.98. The CMJ also showed strong performance in sagittal joints, with hip flexion/extension RMSEs of 6.36° (right) and 5.90° (left), and CMCs of 0.99. Knee flexion/extension during CMJ resulted in RMSEs of 7.34° (right) and 7.06° (left), with CMCs between 0.98 and 0.99. In contrast, the YB tasks showed larger errors and reduced CMCs, particularly in non-sagittal joints. For example, right ankle inversion/eversion RMSEs exceeded 12°, with corresponding CMCs falling as low as 0.21.

#### Within each subgroup

Subgroup-level summaries showed that agreement varied with camera placement, although some between-group differences were small and should be interpreted cautiously relative to commonly used kinematic error thresholds. Front and Back configurations generally showed the most favorable agreement for sagittal-plane angles. For example, right hip flexion/extension RMSE was 4.20° in the Front group and 3.87° in the Back group, with CMC values ≥ 0.99. Similar patterns were observed for knee flexion/extension. In contrast, Same Quadrant configurations showed less favorable agreement, particularly for frontal- and transverse-plane angles. In this group, left hip rotation RMSE reached 15.77° with a CMC of 0.43, while left ankle inversion/eversion RMSE reached 14.76° with a CMC of 0.33.

Diagonal configurations showed intermediate agreement relative to the other subgroups. Although sagittal-plane hip flexion/extension maintained high waveform similarity, RMSE values were higher than those observed in the Front and Back groups, reaching 8.74° and 10.58° for the right and left limbs, respectively, with CMC values of 0.99 and 0.96. Frontal- and transverse-plane angles, particularly hip rotation and ankle inversion/eversion, continued to show reduced agreement.

Left and Right groups showed variable agreement. Sagittal-plane angles, particularly hip and knee flexion/extension, generally maintained low RMSE and high waveform similarity, whereas frontal- and transverse-plane angles showed less consistent agreement. For example, right hip rotation showed RMSE values of 5.56° in the Right group and 6.07° in the Left group, but CMC values differed between groups, indicating that low error magnitude did not always correspond to equivalent waveform similarity.

In summary, the agreement of two-camera joint angle estimation is highly dependent on both the type of movement and the spatial configuration of the cameras (Supplementary Figures S5–S6). Best results were achieved for sagittal plane movements during symmetrical tasks (Squat and CMJ), especially when cameras were placed directly in front of or behind the participant. Same Quadrant configurations showed the least favorable agreement, with higher RMSE and lower waveform similarity, particularly for frontal- and transverse-plane joint angles.

In addition to camera-subgroup agreement metrics provided in Table 2. Task-specific results within each camera subgroup are provided in Supplementary Table S4, with subgroup-specific results presented in separate sheets.

## Discussion

This study evaluated how camera geometry influenced the agreement of three-dimensional lower-limb joint angle estimates from a two-camera markerless motion capture pipeline relative to an eight-camera Theia3D markerless reference system. The main finding was that agreement varied by camera subgroup, joint plane, and task. Overall, Front and Back configurations, which had moderate horizontal angular separation values of 68.59 ± 17.68° and 80.95 ± 22.35°, respectively, generally showed the most favorable agreement, particularly for sagittal-plane joint angles during bilateral tasks. These configurations were captured from cameras positioned at mean heights of 2.43 ± 0.34 m and 2.36 ± 0.39 m, respectively. In contrast, Same Quadrant configurations had the smallest angular separation (28.24 ± 3.81°) and a mean camera height of 2.39 ± 0.19 m, and showed less favorable agreement, particularly for frontal- and transverse-plane joint angles. Diagonal configurations had the largest angular separation (160.56 ± 13.40°) and a mean camera height of 2.39 ± 0.20 m but did not consistently produce better agreement.

Task-specific subgroup results suggest that Front and Back configurations with moderate angular separation are most appropriate for bilateral sagittal-dominant tasks such as squats, countermovement jumps, and broad jumps. Right or Left configurations may be acceptable for unilateral or side-directed tasks when the moving limb remains visible, but their performance should be interpreted cautiously for frontal- and transverse-plane joint angles. Same Quadrant configurations should be avoided where possible because they had the smallest angular separation and consistently showed poorer agreement. Diagonal configurations had the largest angular separation but did not consistently improve agreement, suggesting that excessive separation may reduce shared visibility or introduce task-specific perspective limitations.

### Between systems

Across tasks, joint-specific comparisons revealed that sagittal-plane joint angles, particularly knee flexion/extension, consistently demonstrated greater agreement relative to the 3D baseline, with RMSE values generally below 8° and CMC values approaching 1.00. In contrast, joint angles in the frontal and transverse planes exhibited higher RMSE and lower waveform agreement. A consistent offset was observed in hip flexion/extension angles, with Theia3D systematically yielding lower values across tasks and combinations (RMSE = 17–27°), despite high waveform similarity (CMC > 0.90). This indicates that the larger hip flexion/extension error was driven primarily by systematic angular offset rather than poor temporal agreement or waveform shape. Similar offsets have been reported in previous comparisons between markerless and marker-based systems, with differences of approximately 11° during gait (4, 18, 19) and 6.7–13.8° during dynamic athletic tasks (20). These discrepancies may be attributed to differences in pelvis segment definitions between systems, with Theia3D adopting a more neutral pelvic orientation, as described in Theia3D's technical documentation. Although the current two-camera pipeline employed a markerless workflow, the LSTM-based marker augmentation algorithm was trained primarily on optical marker data, which may have contributed to systematic differences. Additionally, variation in pose estimation models (e.g., RTMPose vs. Theia3D's proprietary model) likely introduced further divergence in these joint angle outputs.

Task-specific results further demonstrated that camera-geometry effects were not uniform across movement types. Bilateral sagittal-dominant tasks, such as squats and countermovement jumps, showed more consistent agreement across camera groups. In contrast, Y-balance showed poorer agreement, likely because the task involves lower-limb crossing, quasi-static control, and frequent self-occlusion of distal segments. L-hop also showed greater variability, but manual verification indicated that many failed L-hop trials occurred because participants moved partially or fully outside the field of view of one or both cameras. These findings emphasize that reduced-camera markerless systems should be evaluated not only by agreement metrics from successful trials, but also by trial-retention rate and task-specific robustness. Previous studies validating markerless motion capture systems like OpenCap have primarily focused on dynamic, non-crossing movements such as single-leg hops, drop jumps, and gait, and have not typically included complex tasks like YB that involve limb crossing (8, 9).

Overall, Front and Back configurations showed the most favorable agreement during bilateral sagittal-dominant tasks, whereas Left and Right configurations showed more variable performance across joints and planes. Although Left and Right groups maintained favorable sagittal-plane agreement, frontal- and transverse-plane angles showed greater variability, particularly for hip rotation and ankle inversion/eversion. Same Quadrant configurations showed less favorable agreement, consistent with their small horizontal angular separation (28.24 ± 3.81°), which likely reduced perspective diversity for three-dimensional reconstruction. Diagonal configurations had the largest angular separation (160.56 ± 13.40°), but did not consistently improve agreement, suggesting that large separation alone was insufficient when task-specific segment visibility or shared coverage was limited. These findings are consistent with prior two-camera studies in which cameras were commonly positioned anterior to the participant and angled approximately 45–60° from the sagittal axis to support depth reconstruction while maintaining visibility of the moving segments (7–9, 11).

In addition, differences in joint coordinate definitions likely contributed to the ankle discrepancies observed between systems. Theia3D reports ankle rotations using three orthogonal Cartesian axes, whereas *Model_Pose2Sim_Halpe26.osim* represents the ankle as a CustomJoint with a non-Cartesian flexion axis and represents subtalar motion separately as a PinJoint. Therefore, ankle plantarflexion/dorsiflexion and inversion/eversion differences may partly reflect model-definition differences rather than only reconstruction error.

### Within combinations

Across all tasks and camera combinations, joint angles in the sagittal plane demonstrated the most favorable agreement, with median RMSE values below 10 degrees and very strong waveform similarity as reflected by high median CMC values (Table 3). In contrast, ankle inversion/eversion consistently yielded the lowest median CMC values. This result aligns with prior research suggesting that foot tracking remains a persistent challenge in markerless motion capture systems using two-camera configurations (4). Indeed, foot joint angles have historically shown the poorest accuracy in both markerless and marker-based systems, largely due to the complex anatomy and dynamic behavior of the foot, which require dense marker sets for precise tracking (21).

Task- and joint-specific patterns further indicated that camera-geometry effects were not uniform across all movements. Although Front and Back configurations showed broadly favorable agreement overall, some Back-group combinations showed higher ankle plantar-/dorsi-flexion and inversion/eversion errors during Y-balance. This likely reflects the interaction between camera placement and task-specific distal-segment visibility. During Y-balance, the reaching limb crosses or moves away from the stance limb, increasing the likelihood of foot occlusion, reduced keypoint confidence, and poorer triangulation of distal segments. In contrast, sagittal-dominant bilateral tasks such as squats and countermovement jumps maintained more consistent foot visibility and produced lower RMSE across most camera groups. Therefore, camera-geometry effects should be interpreted jointly with task demands and joint segment visibility, rather than as a uniform group-level effect.

Squat movements, which involve fixed foot positions and largely sagittal-plane motion (e.g., plantarflexion/dorsiflexion), showed good agreement in nearly all lower limb joints bilaterally, as illustrated in Figure 5. The reduced agreement observed at the ankle may reflect the combined effects of camera geometry, including angular separation, field of view, viewing perspective, and task-specific visibility of the distal segments. In the present setup, camera height averaged 2.39 ± 0.36 m, while horizontal angular separation varied substantially across camera subgroups, from 28.24 ± 3.81° in Same Quadrant configurations to 160.56 ± 13.40° in Diagonal configurations. Therefore, ankle errors are unlikely to be explained by camera height alone. This interpretation is also supported by (22), who reported comparable RMSE values between truss-mounted cameras at 3 m and tripod-mounted cameras at 1.4 m during jumping tasks. Accordingly, the ankle findings should be interpreted as reflecting broader distal-segment tracking challenges in reduced-camera markerless reconstruction, particularly when camera geometry limits foot visibility or depth resolution.

Unlike single-camera systems that are susceptible to severe viewpoint constraints, two-camera setups provide a balanced trade-off between ease of deployment and biomechanical fidelity (10). Importantly, the ability to capture detailed biomechanical data during context-specific movements, such as passing, shooting, or dynamic balance tasks, could greatly enhance individualized injury risk profiling and real-time intervention strategies (23, 24).

### Limitations

Several limitations should be acknowledged. First, the eight-camera Theia3D system served as a high-quality markerless reference, but it should not be interpreted as a definitive biomechanical gold standard. Therefore, the reported between-system results reflect agreement with Theia3D rather than absolute kinematic accuracy. In addition, some discrepancies may partly reflect differences in model definitions and joint coordinate conventions. Future work should validate reduced-camera workflows directly against marker-based optical motion capture systems.

Second, camera-to-participant distance, focal length/zoom, field of view, lighting, clothing, and task-specific segment visibility may all have contributed to configuration-dependent differences. Accordingly, subgroup effects should be interpreted as combined camera-configuration effects rather than the isolated influence of angular separation alone.

Third, the two-camera workflow remains sensitive to occlusion, field-of-view loss, and limited segment visibility, particularly for frontal/transverse-plane motions and tasks involving limb crossing or quasi-static control, such as Y-Balance. These limitations emphasize the need for task-specific camera placement and careful interpretation of distal-segment kinematics.

Finally, the findings are specific to the RTMPose—Pose2Sim—LSTM—OpenSim workflow and may not generalize to other markerless systems. Camera orientation effects can be algorithm- and task-dependent, as shown in OpenPose/OpenCap-based gait analyses (25). The sample also consisted only of healthy female soccer athletes, limiting generalizability to male athletes, youth athletes, older adults, clinical populations, and individuals with altered movement patterns.

## Conclusion

From an applied perspective, camera placement should be selected according to the movement task and joint angles of interest. Front and Back configurations, with moderate horizontal angular separation values of 68.59 ± 17.68° and 80.95 ± 22.35°, generally showed more favorable agreement for sagittal-dominant bilateral tasks such as squats, countermovement jumps, and broad jumps. Same Quadrant configurations had the smallest angular separation (28.24 ± 3.81°) and should be avoided where possible, especially for frontal- and transverse-plane joint angles. Diagonal configurations had the largest angular separation (160.56 ± 13.40°), but did not consistently improve agreement, indicating that angular separation alone is insufficient. For tasks involving limb crossing or large displacement, such as Y-balance and L-hop, camera placement should prioritize continuous visibility of the foot and ankle and full coverage of the movement path.

## Data Availability

De-identified processed data may be made available upon reasonable request, subject to ethics approval and participant consent.
