# Supplementary figures and images for: Influence of camera geometry on 3D joint angle estimation in markerless motion capture
Source: Front Sports Act Living. 2026 Jul 13;8:1850018. doi: 10.3389/fspor.2026.1850018 (PMC13402369; doi:10.3389/fspor.2026.1850018)

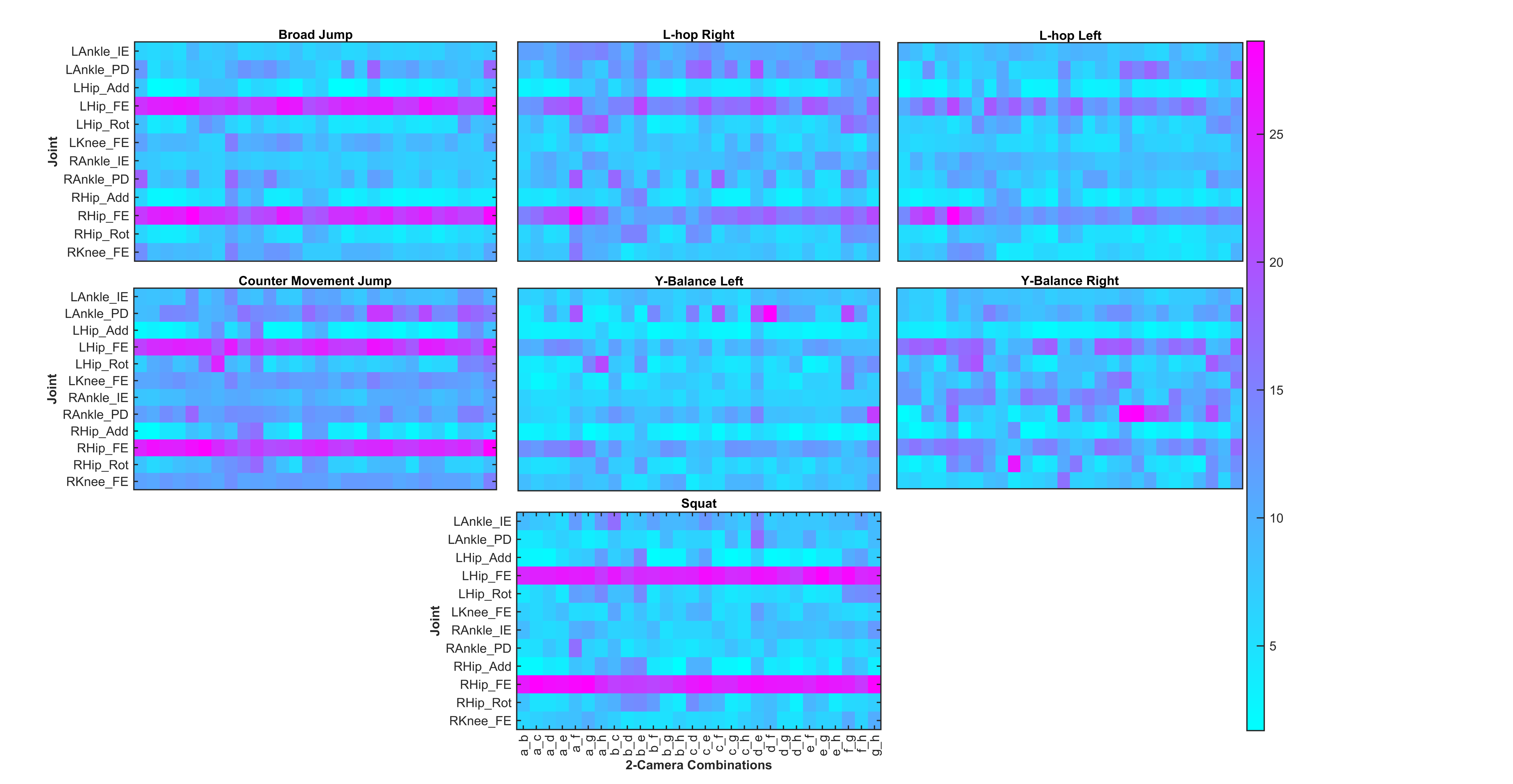

Supplement: Supplementary file 1 [file Image1.tif]

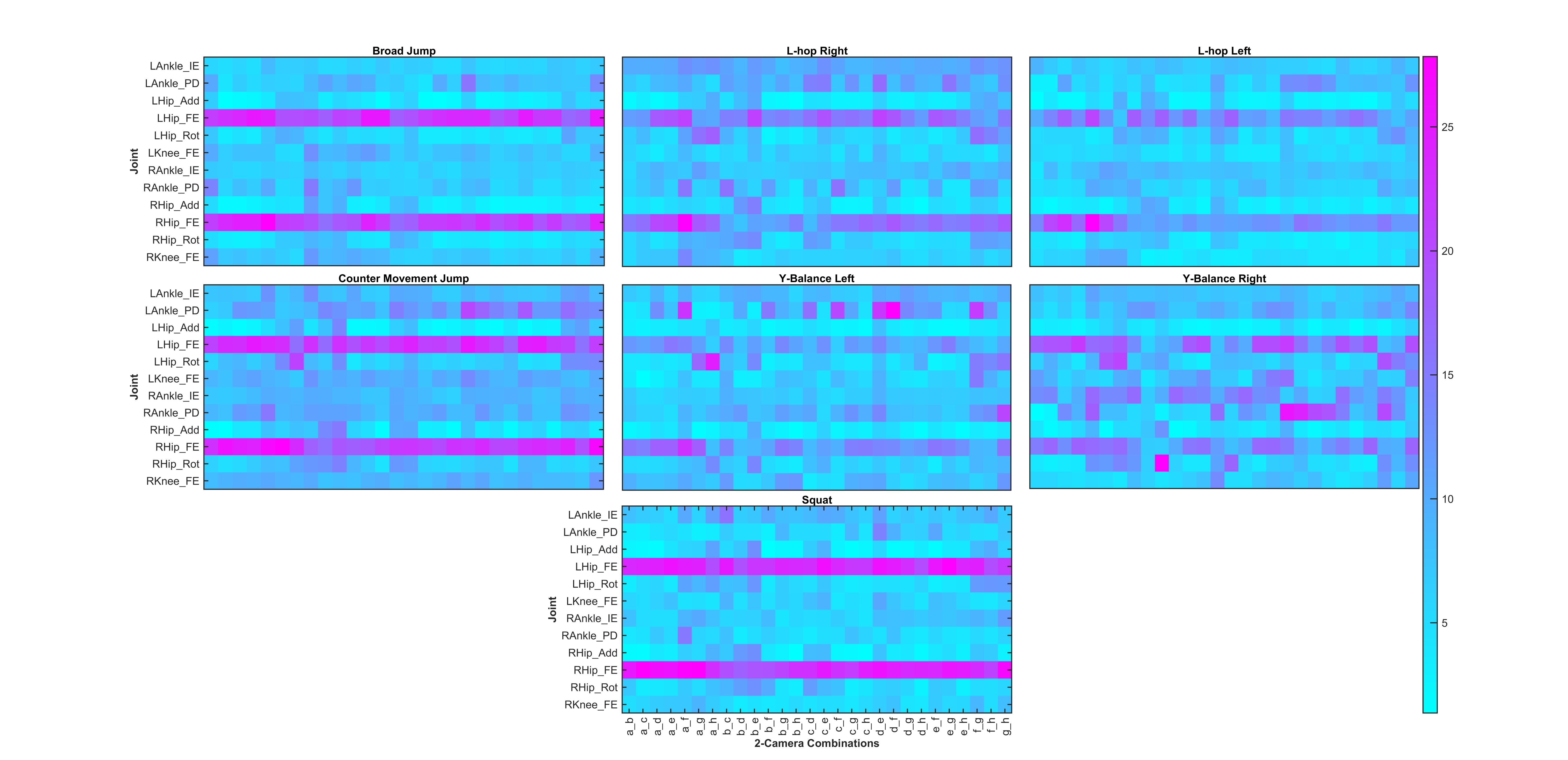

Supplement: Supplementary file 2 [file Image2.tif]

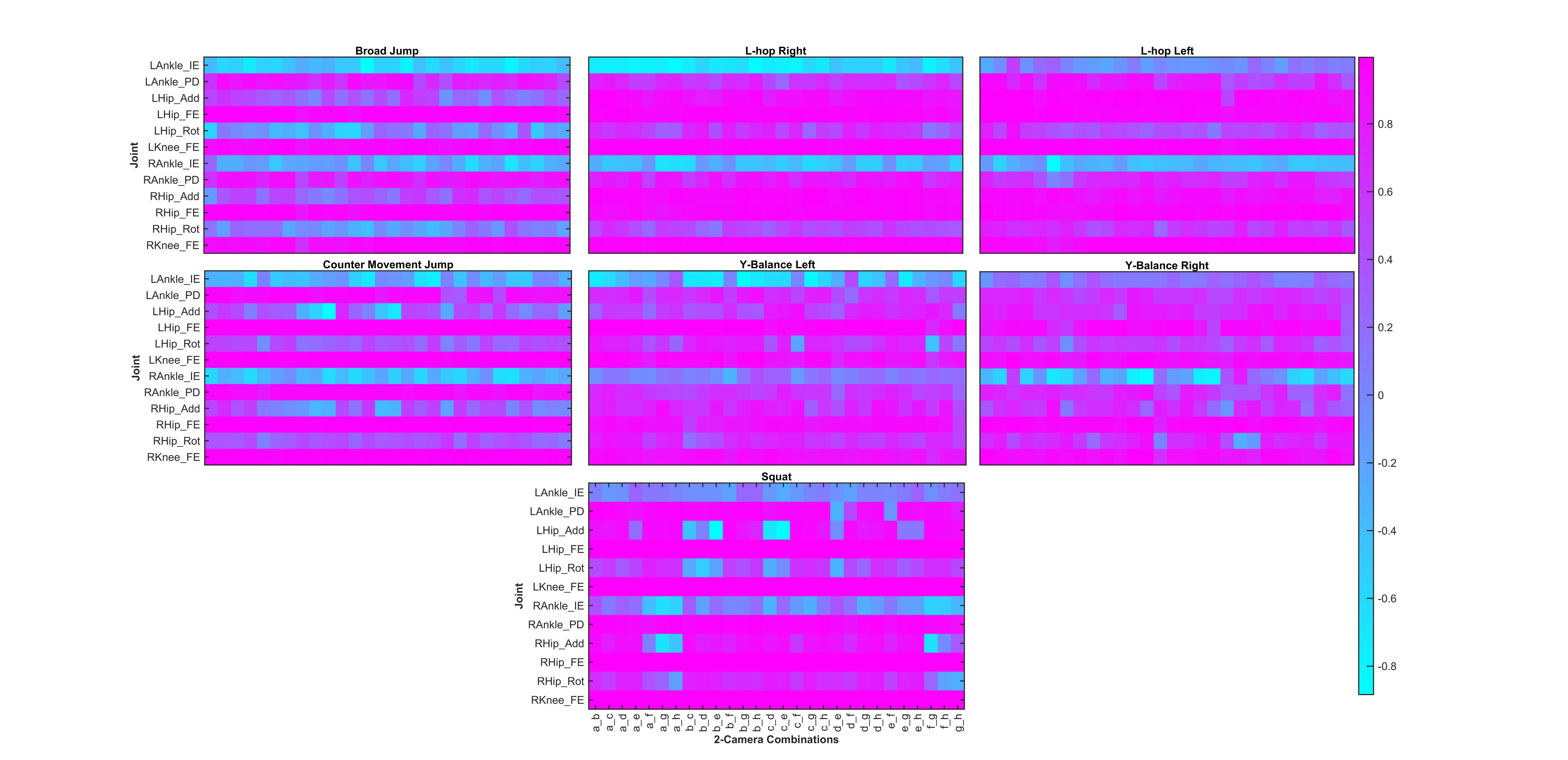

Supplement: Supplementary file 3 [file Image3.tif]

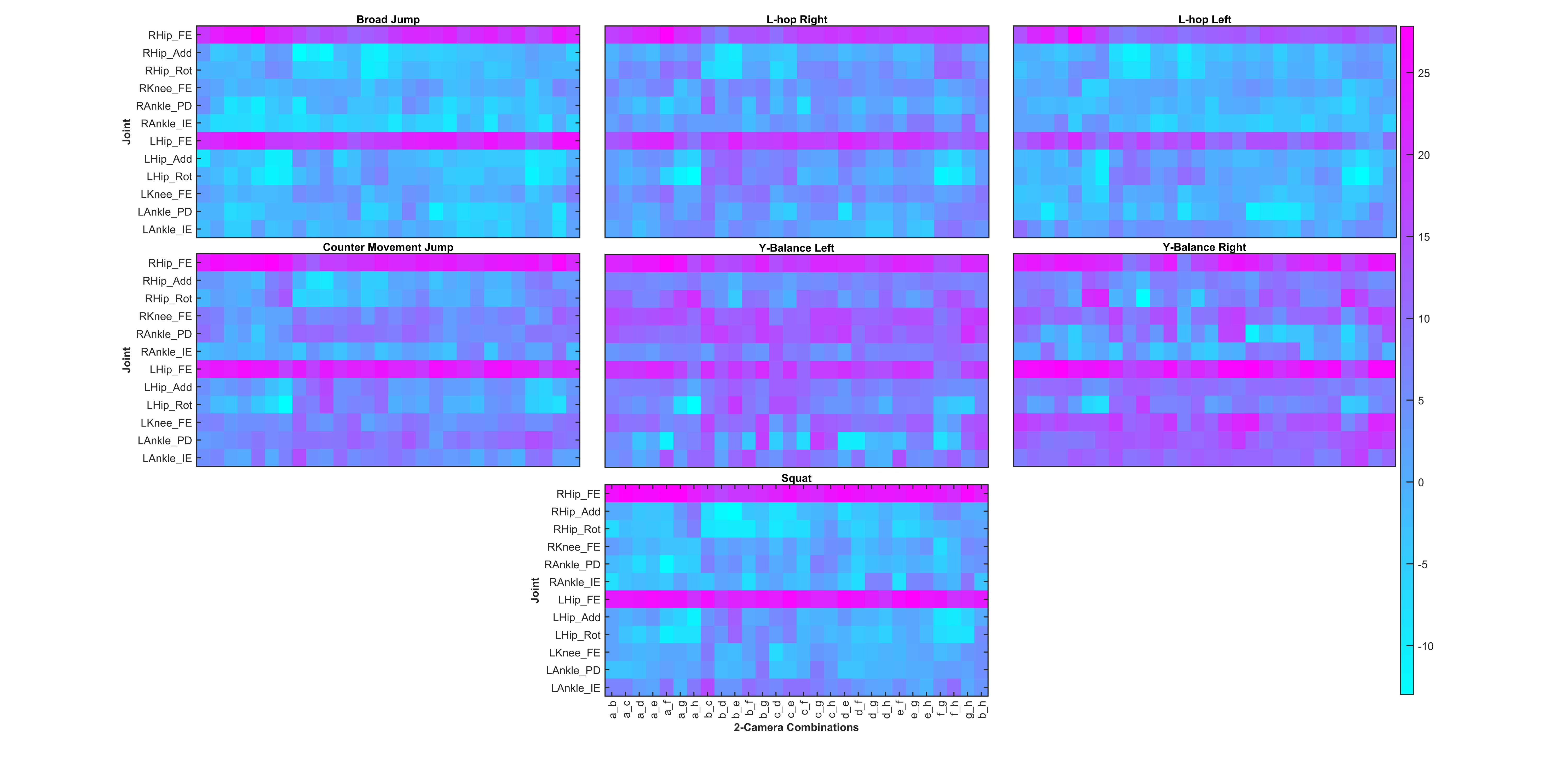

Supplement: Supplementary file 4 [file Image4.tif]

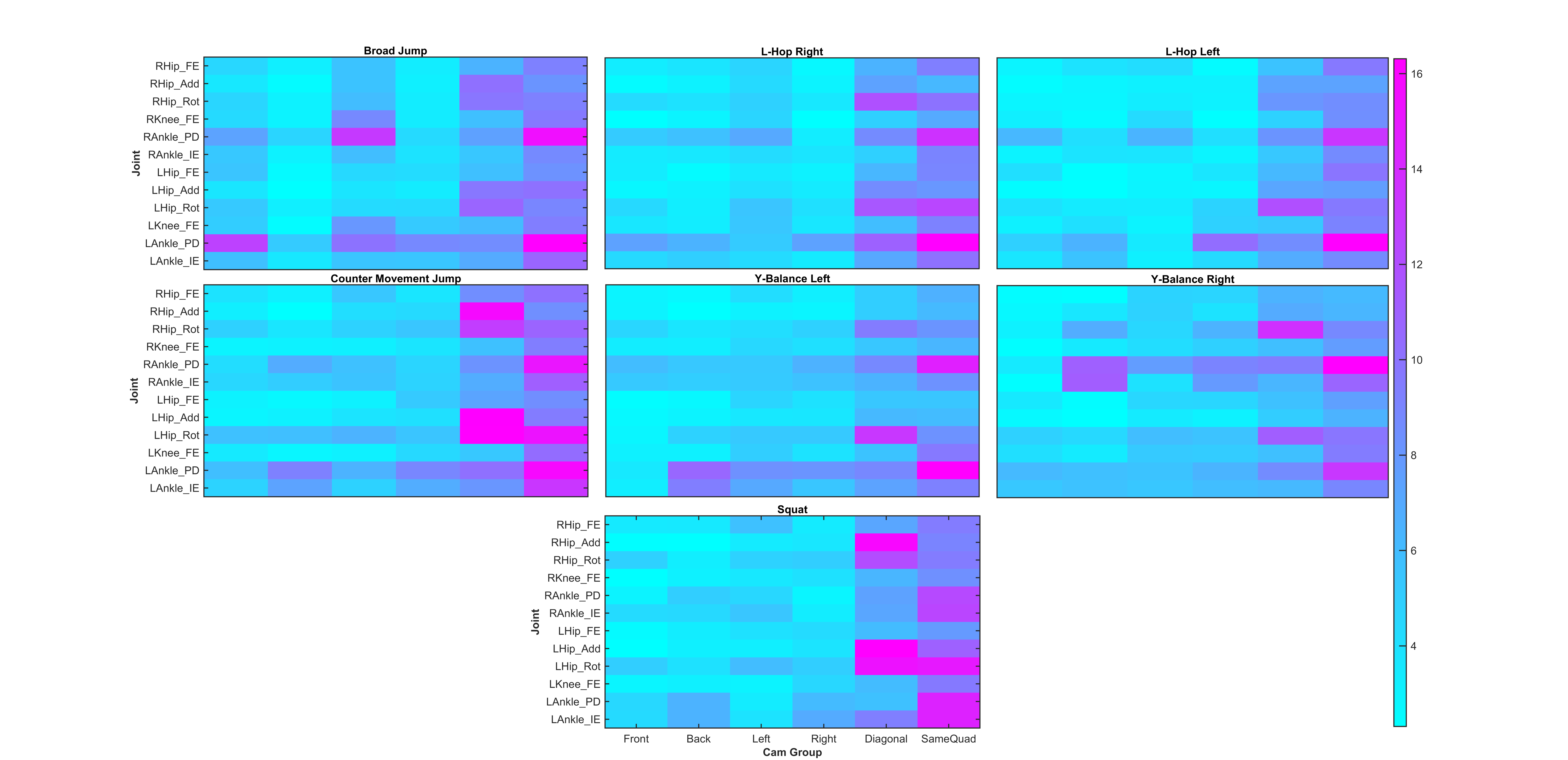

Supplement: Supplementary file 5 [file Image5.tif]

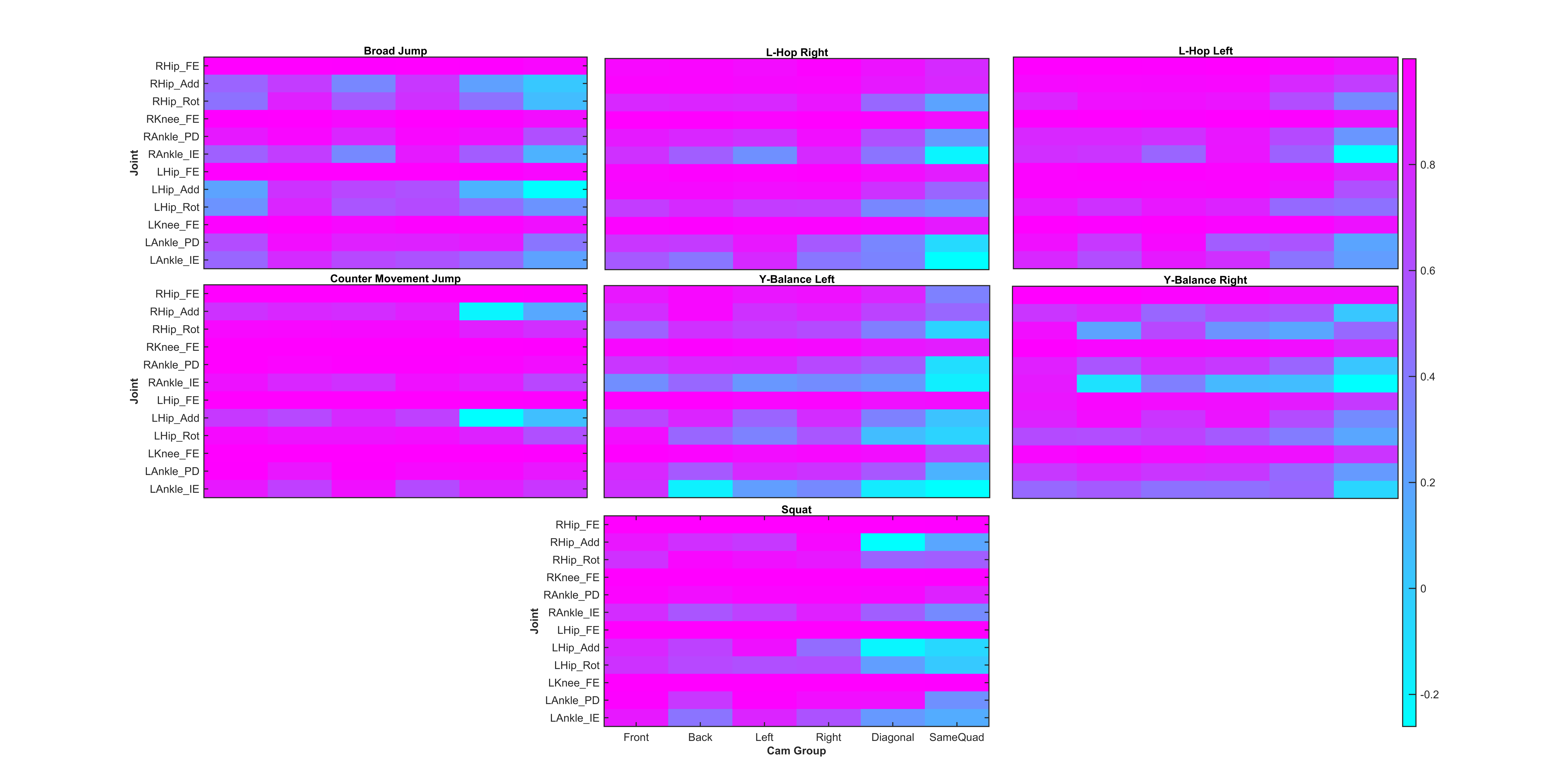

Supplement: Supplementary file 6 [file Image6.tif]
