# Supplementary material for: Influence of camera geometry on 3D joint angle estimation in markerless motion capture
Source: Front Sports Act Living. 2026 Jul 13;8:1850018. doi: 10.3389/fspor.2026.1850018 (PMC13402369; doi:10.3389/fspor.2026.1850018)
Supplement: Supplementary file 7 [file Datasheet1.docx]

# **Supplementary material 1**

Table S1 defines the camera-combination subgroups based on the horizontal spatial arrangement of each two-camera pair and reports the corresponding horizontal angular separation for each combination. The camera labels (a-h) correspond to the calibrated camera locations shown in Figure 1.

**Table S1:** Camera-combination subgroup definitions and horizontal angular separation.

| **Group** | **Combination** | **Angular Separation (deg)** | **Mean±SD Angular Separation (deg)** |
| --- | --- | --- | --- |
| Front | a_e | 93.46 | 68.59±17.68 |
|  | a_d | 71.11 |  |
|  | f_e | 66.06 |  |
|  | f_d | 43.72 |  |
| Back | b_h | 112.55 | 80.95±22.35 |
|  | b_g | 81.05 |  |
|  | c_h | 80.84 |  |
|  | c_g | 49.34 |  |
| Left | d_h | 111.30 | 115.88±19.31 |
|  | d_g | 142.81 |  |
|  | e_h | 88.96 |  |
|  | e_g | 120.46 |  |
| Right | f_b | 92.42 | 94.58±20.95 |
|  | f_c | 124.13 |  |
|  | a_b | 65.03 |  |
|  | a_c | 96.74 |  |
| Same quadrant | a_f | 27.39 | 28.24±3.81 |
|  | e_d | 22.34 |  |
|  | b_c | 31.71 |  |
|  | g_h | 31.50 |  |
| Diagonal | c_d | 167.85 | 160.56±13.40 |
|  | c_e | 169.80 |  |
|  | b_d | 136.14 |  |
|  | b_e | 158.49 |  |
|  | a_g | 146.08 |  |
|  | a_h | 177.58 |  |
|  | f_g | 173.47 |  |
|  | f_h | 155.03 |  |

Note: Front: Both cameras in the front half (left-front and right-front); Back: Both cameras in the back half (left-back and right-back); Left: One camera in the left-back and one in the left-front; Right: One camera in the right-back and one in the right-front; Diagonal: Cameras positioned diagonally across opposite quadrants (left-back & right-front or left-front & right-back); Same quadrant: Both cameras located within the same quadrant. Angular separation was defined as the horizontal angle between the two camera-center position vectors projected onto the laboratory floor plane. Values are reported in degrees. The final column reports the mean ± standard deviation of angular separation within each subgroup.

Table S2 summarizes the intrinsic and extrinsic calibration parameters for each camera, including focal length, camera height, distance to the laboratory origin, azimuth angle, and estimated horizontal and vertical field of view. The camera labels (a-h) correspond to the calibrated camera locations shown in Figure 1.

**Table S2:** Camera intrinsic and extrinsic parameters derived from the calibration file.

| **Camera** | **Focal Length (px)** | **Height (mm)** | **Horizontal Distance to Origin (mm)** | **Distance to Origin (mm)** | **Azimuth (deg)** | **Horizontal FOV (deg)** | **Vertical FOV (deg)** |
| --- | --- | --- | --- | --- | --- | --- | --- |
| a | 1318.04 | 1809.54 | 3651.73 | 4075.48 | 36.46 | 72.14 | 44.56 |
| b | 1264.12 | 1797.29 | 3509.40 | 3942.94 | -28.57 | 74.43 | 46.26 |
| c | 1700.11 | 2556.70 | 4699.22 | 5349.71 | -60.28 | 58.90 | 35.24 |
| d | 1826.64 | 2560.13 | 4747.08 | 5393.43 | 107.58 | 55.45 | 32.94 |
| e | 1503.91 | 2593.48 | 3474.20 | 4335.46 | 129.92 | 65.10 | 39.50 |
| f | 1780.65 | 2656.90 | 5112.27 | 5761.46 | 63.85 | 56.66 | 33.74 |
| g | 1278.60 | 2589.94 | 4381.07 | 5089.36 | -109.62 | 73.80 | 45.79 |
| h | 1273.94 | 2551.18 | 2775.50 | 3769.87 | -141.12 | 74.00 | 45.94 |

**Note.** All parameters were extracted from the calibration file or calculated from the extracted intrinsic and extrinsic camera parameters. **Focal Length (px):** calibrated camera focal length expressed in pixels. This reflects the effective zoom/framing of each camera; **Height (mm):** vertical position of the camera center relative to the laboratory/world coordinate system; **Horizontal Distance to Origin (mm):** floor-plane distance from the camera center to the world origin, calculated using only the X and Y coordinates: sqrt(X^2 + Y^2); **Distance to Origin (mm):** three-dimensional distance from the camera center to the world origin, calculated using X, Y, and Z coordinates: sqrt(X^2 + Y^2 + Z^2); **Azimuth (deg):** horizontal angular position of each camera around the world origin, calculated from the camera X-Y coordinates using atan2(Y, X); **Horizontal FOV (deg):** estimated horizontal field of view, calculated from image width and focal length: 2 x tan^-1(image width / [2 x focal length]); **Vertical FOV (deg):** estimated vertical field of view, calculated from image height and focal length: 2 x tan^-1(image height / [2 x focal length]).

Table S3 summarizes the statistical comparison of camera-geometry subgroup characteristics. Pair-level subgroup differences were examined for focal length, camera height, distance -to-origin, field of view, and angular separation. Focal length, camera height, distance-to-origin, and horizontal/vertical field of view did not differ meaningfully between subgroups, whereas horizontal angular separation differed across subgroups.

**Table S3**: Statistical comparison of camera-geometry characteristics across subgroups.

| **Variable** | **Between-group difference** | **ANOVA p-value** | **Kruskal–Wallis p-value** |
| --- | --- | --- | --- |
| Focal length | No | *p* = 0.482 | *p* = 0.322 |
| Camera height | No | *p* = 0.397 | *p* = 0.622 |
| Horizontal distance to origin | No | *p* = 0.802 | *p* = 0.712 |
| 3D distance to origin | No | *p* = 0.953 | *p* = 0.839 |
| Horizontal FOV | No | *p* = 0.456 | *p* = 0.319 |
| Vertical FOV | No | *p* = 0.448 | *p* = 0.319 |
| Horizontal angular separation | Yes | *p* < 0.001 | *p* < 0.001 |

Note: Values represent one-way ANOVA and Kruskal–Wallis test results comparing camera-geometry subgroups. Variables included focal length, camera height, horizontal and three-dimensional distance-to-origin, horizontal and vertical field of view, and horizontal angular separation. One-way ANOVA was used as the primary comparison, and Kruskal–Wallis tests were used as non-parametric sensitivity analyses. FOV = field of view; 3D = three-dimensional. p < 0.05 was considered evidence of a subgroup difference.

Supplementary Figures S1-28 (Front S1-4; Back S5-8; Left S9-12; Right S13-16; Same quadrant S17-20; Diagonal S21-28) provide pair-specific top-view visualizations of all two-camera configurations evaluated in this study. Each plot displays the calibrated camera-center locations relative to the laboratory origin, with the selected camera pair highlighted and all non-selected cameras shown in grey for spatial reference. The horizontal axis represents the anterior-posterior direction, and the vertical axis represents the medial-lateral direction. Solid colored lines indicate the projected camera-center vectors from the origin to each selected camera, while the dashed black line represents the horizontal baseline between the two cameras. The black arc indicates horizontal angular separation, calculated as the angle between the two camera-center vectors projected onto the laboratory floor plane. Shaded sectors represent the estimated horizontal field of view (FOV) of each selected camera, calculated from calibrated focal length and image resolution and oriented toward the capture origin for visualization.

**Front subgroup (Figures S1-S4):**


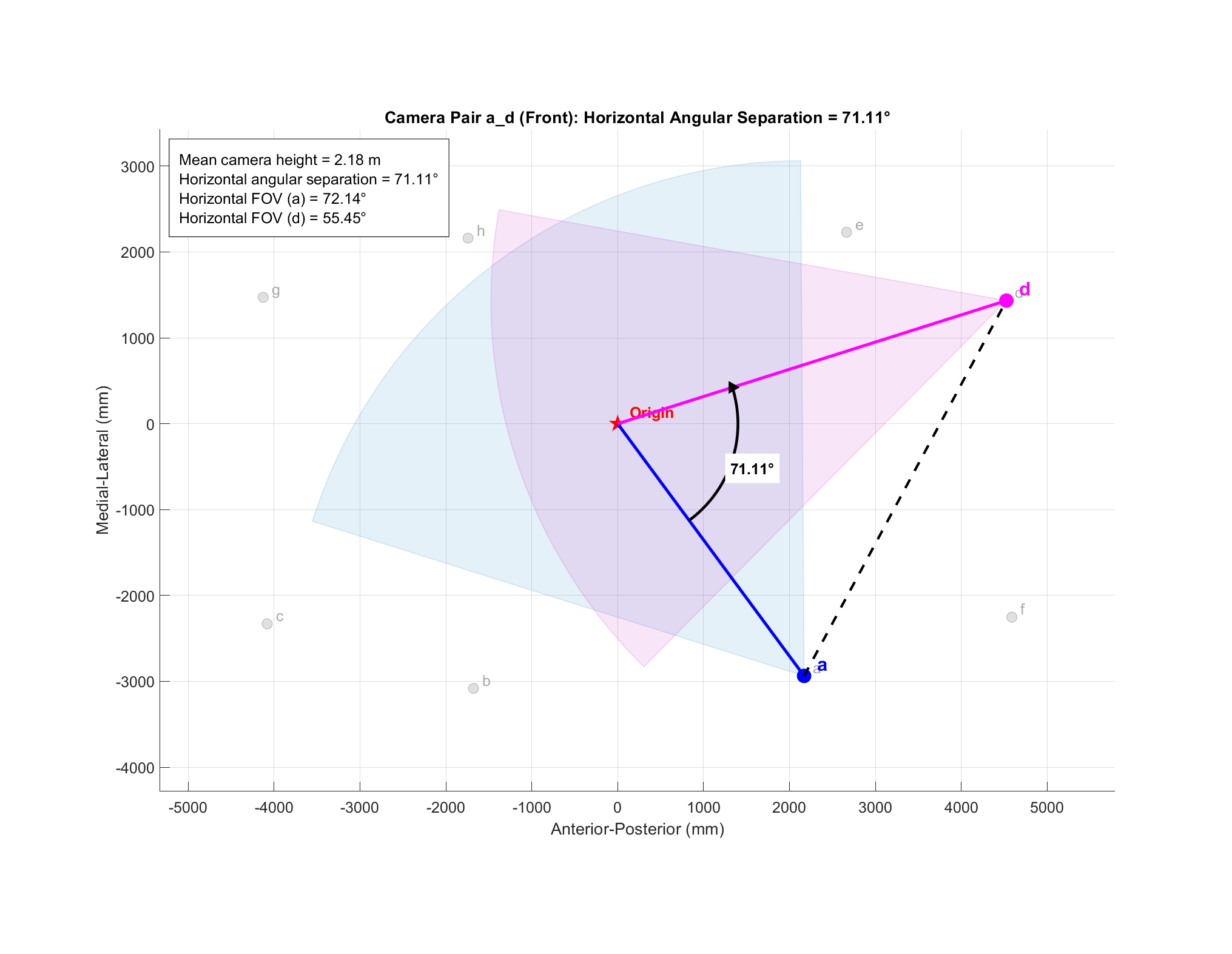


**Figure S1:** Camera pair a_d. Mean camera height was 2.18 m, horizontal angular separation was 71.11°, and horizontal field of view was 72.14° for camera **a** and 55.45° for camera **d**.


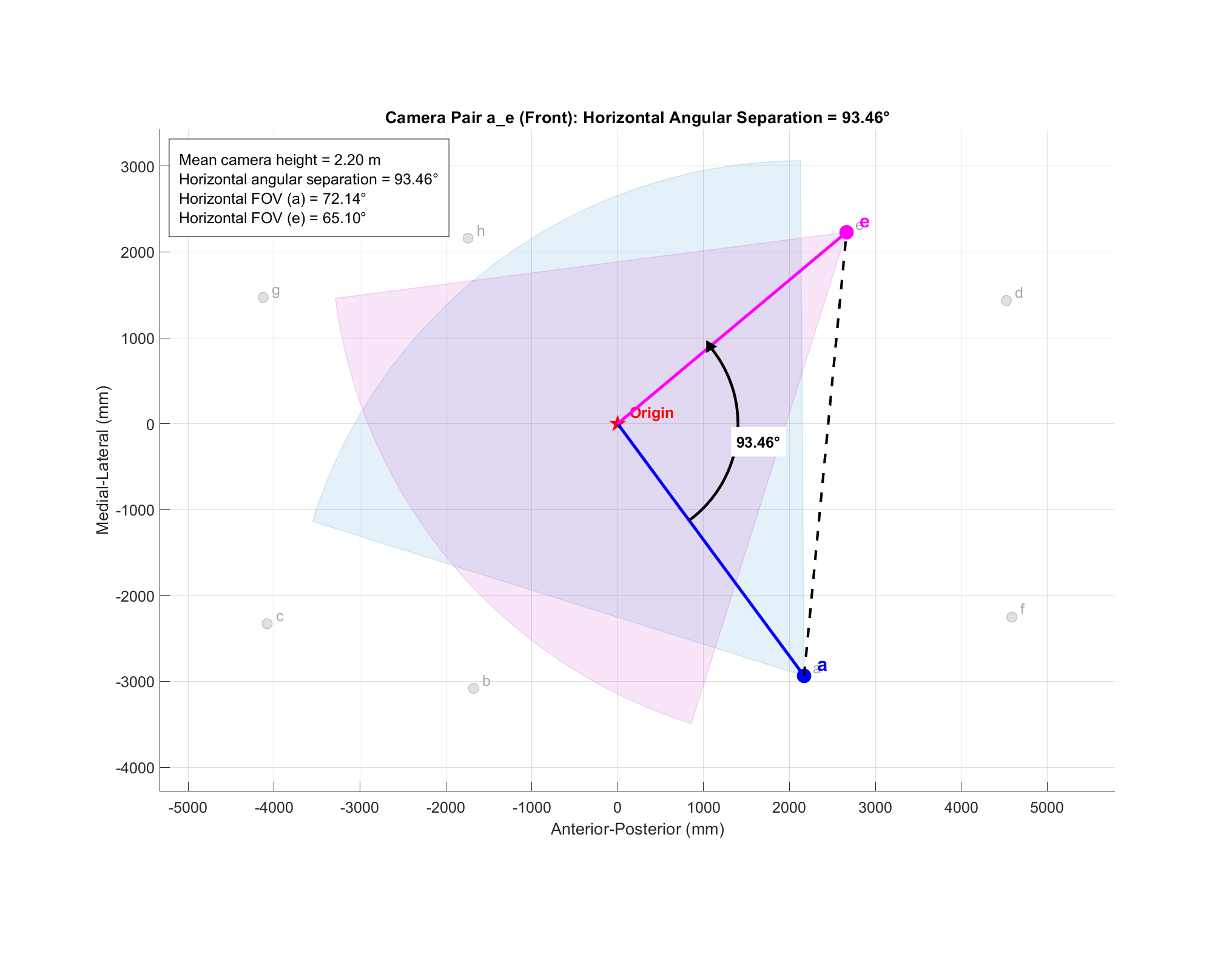


**Figure S2:** Camera pair a_e. Mean camera height was 2.20 m, horizontal angular separation was 93.46°, and horizontal field of view was 72.14° for camera **a** and 65.10° for camera **e**.


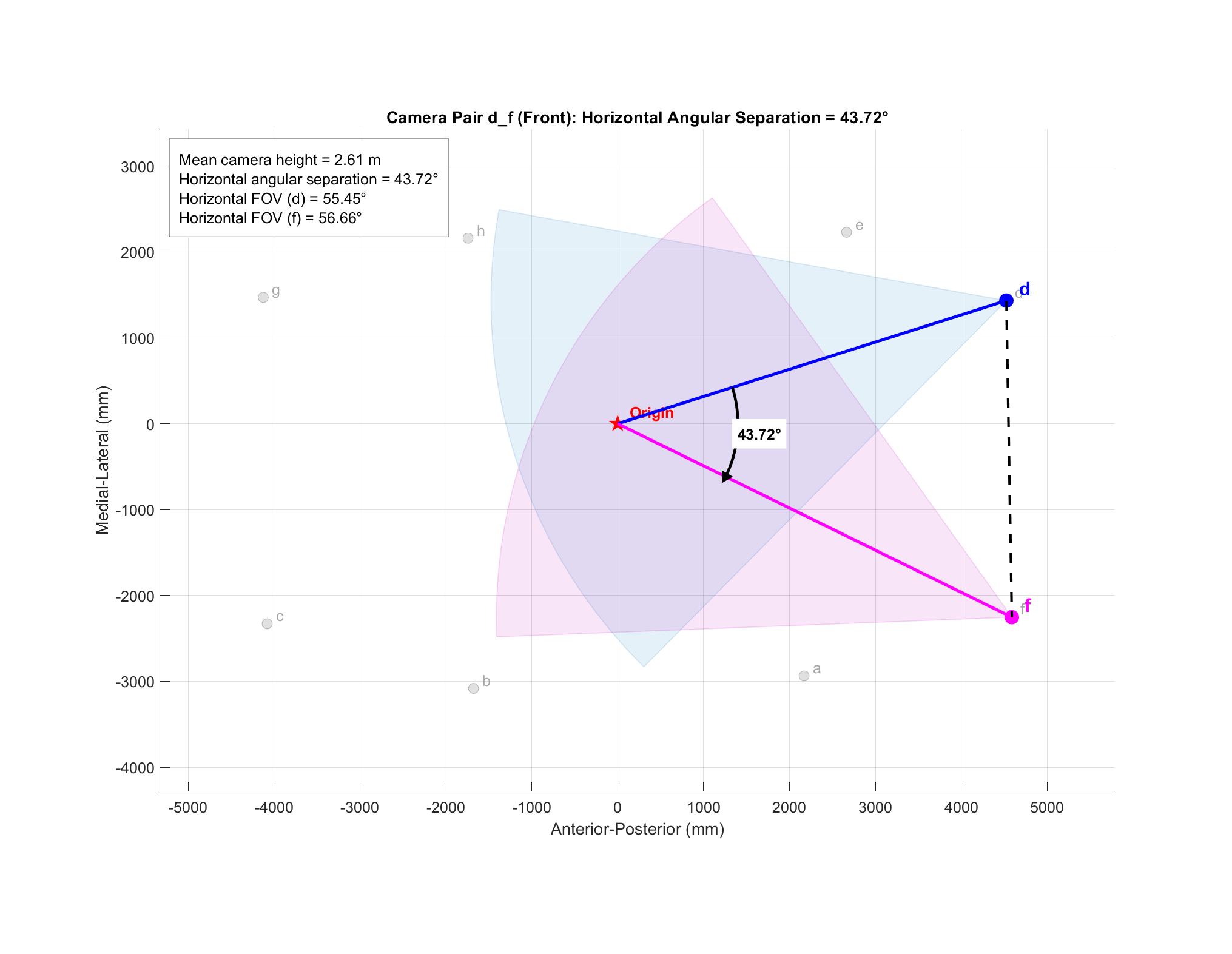


**Figure S3:** Camera pair d_f. Mean camera height was 2.61 m, horizontal angular separation was 43.72°, and horizontal field of view was 55.45° for camera **d** and 56.66° for camera **f**.


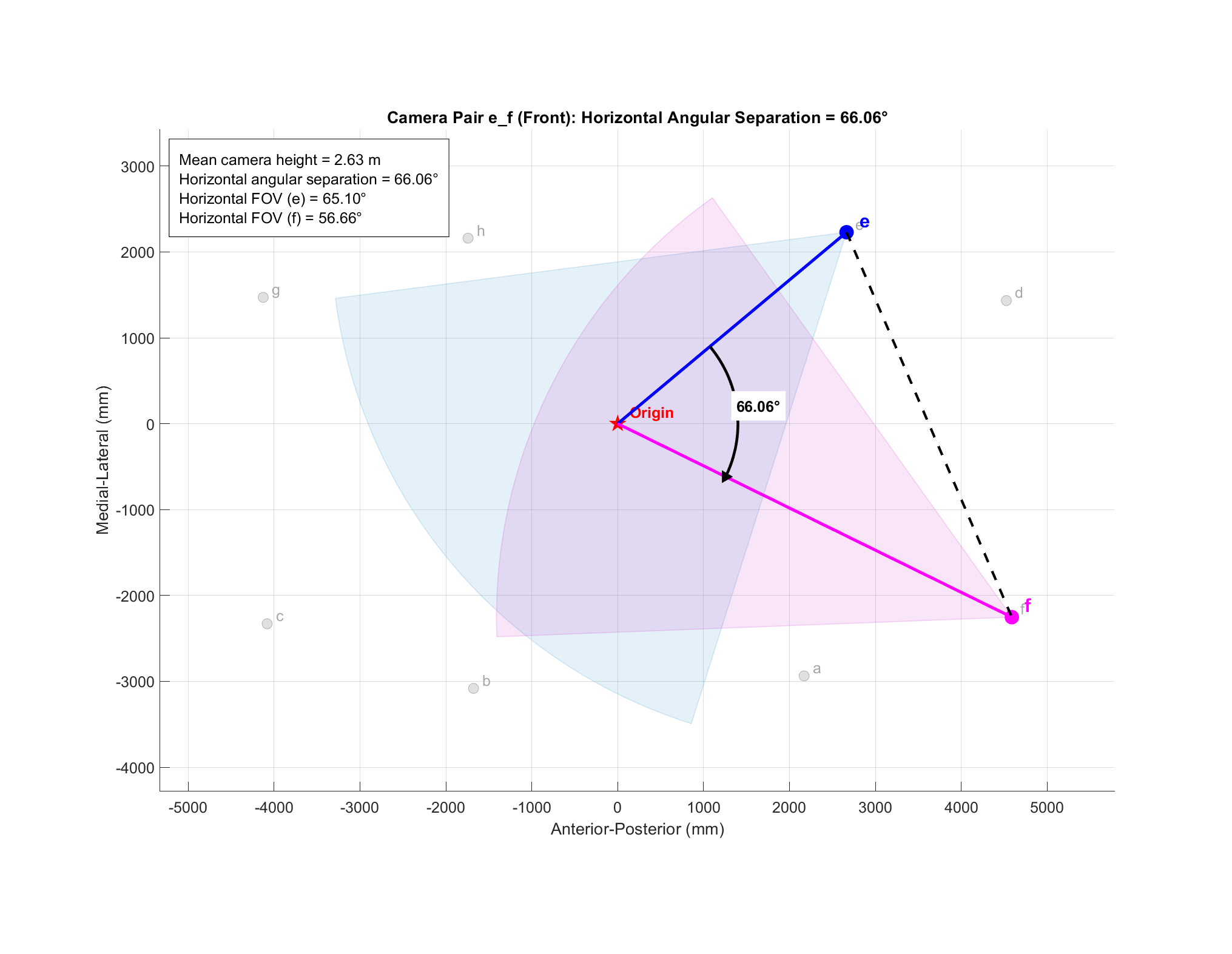


**Figure S4:** Camera pair e_f. Mean camera height was 2.63 m, horizontal angular separation was 66.06°, and horizontal field of view was 65.10° for camera **e** and 56.66° for camera **f**.

**Back subgroup (Figures S5-S8):**


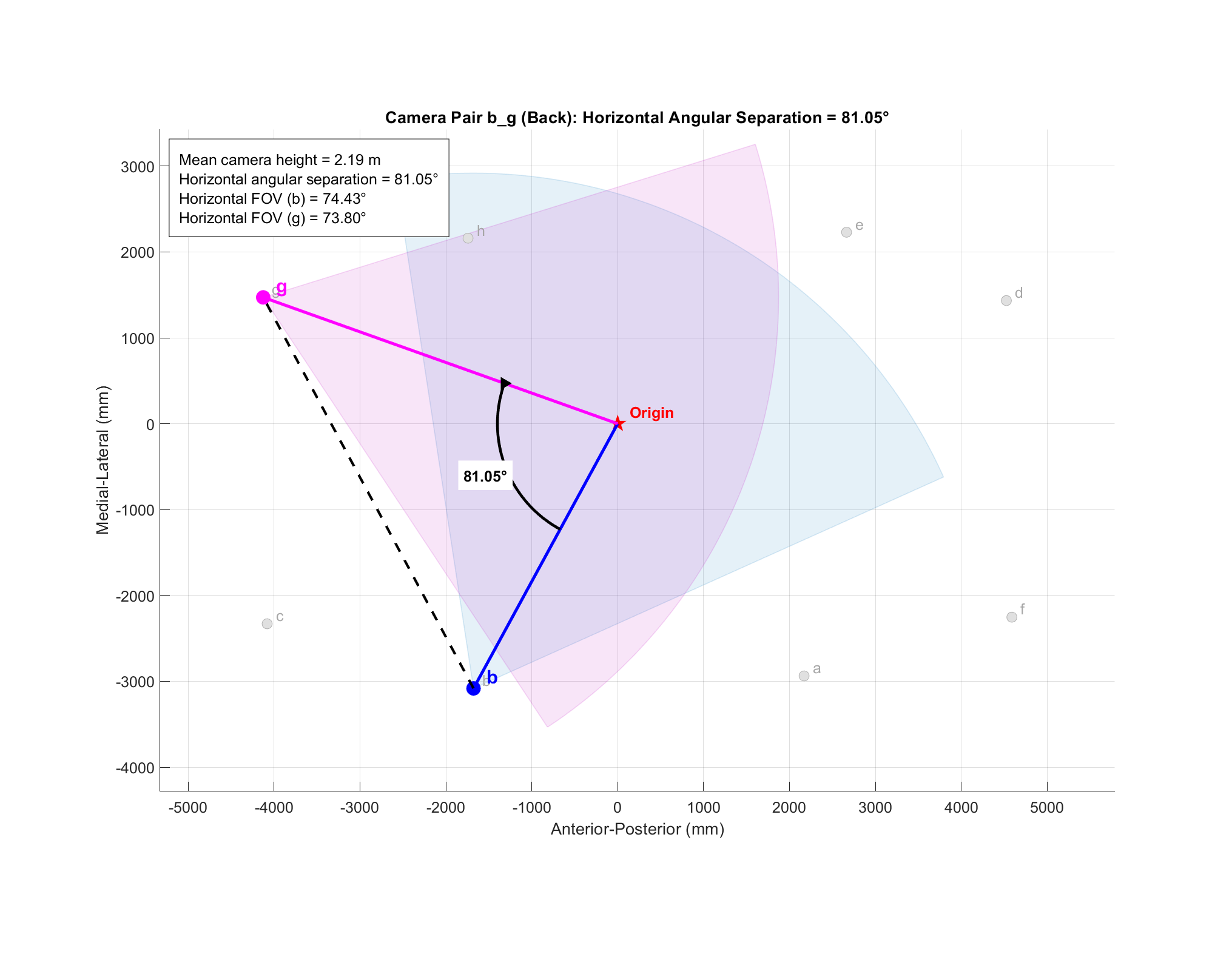


**Figure S5:** Camera pair b_g. Mean camera height was 2.19 m, horizontal angular separation was 81.05°, and horizontal field of view was 74.43° for camera **b** and 73.80° for camera **g**.


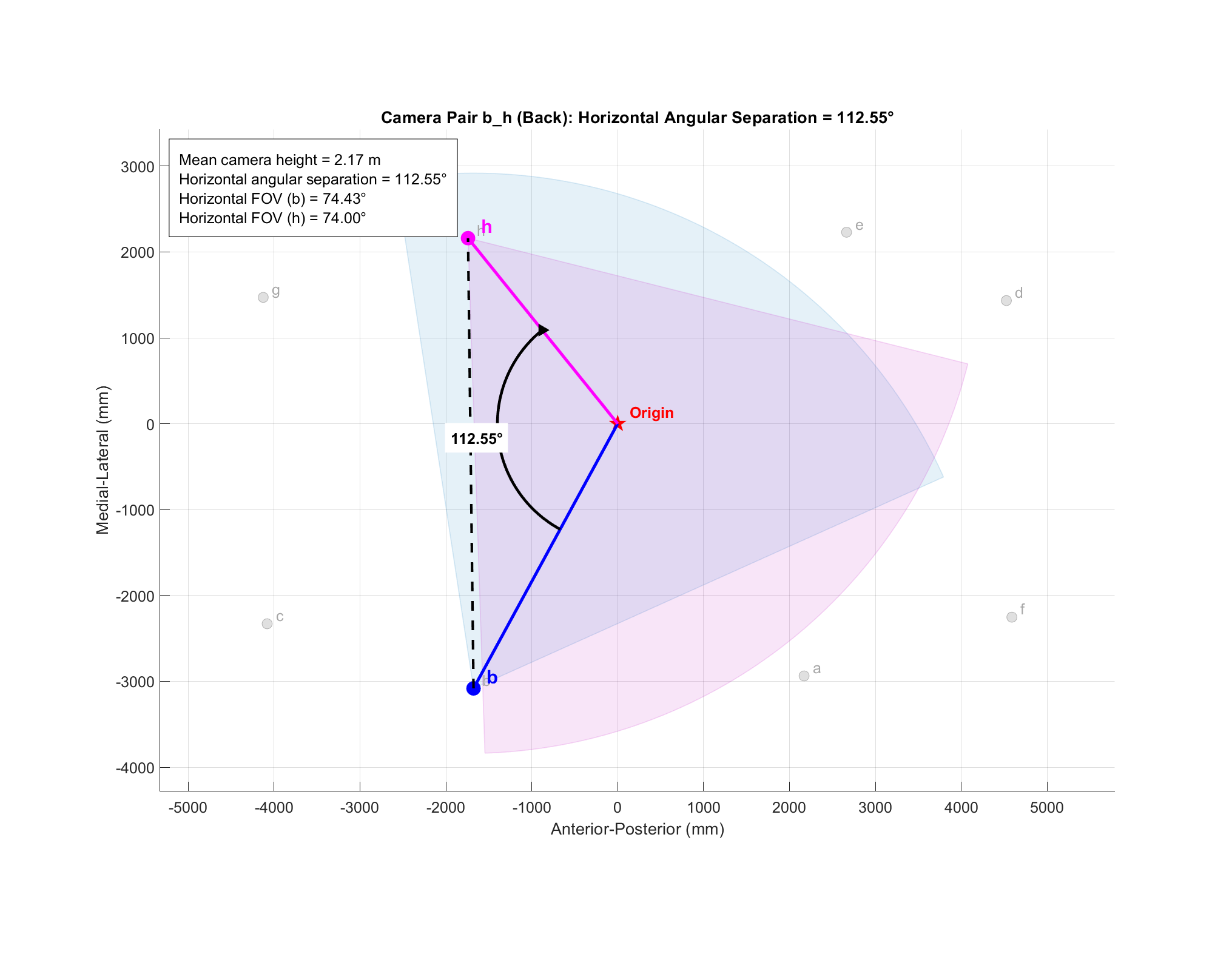


**Figure S6:** Camera pair b_h. Mean camera height was 2.17 m, horizontal angular separation was 112.55°, and horizontal field of view was 74.43° for camera **b** and 74.00° for camera **h**.


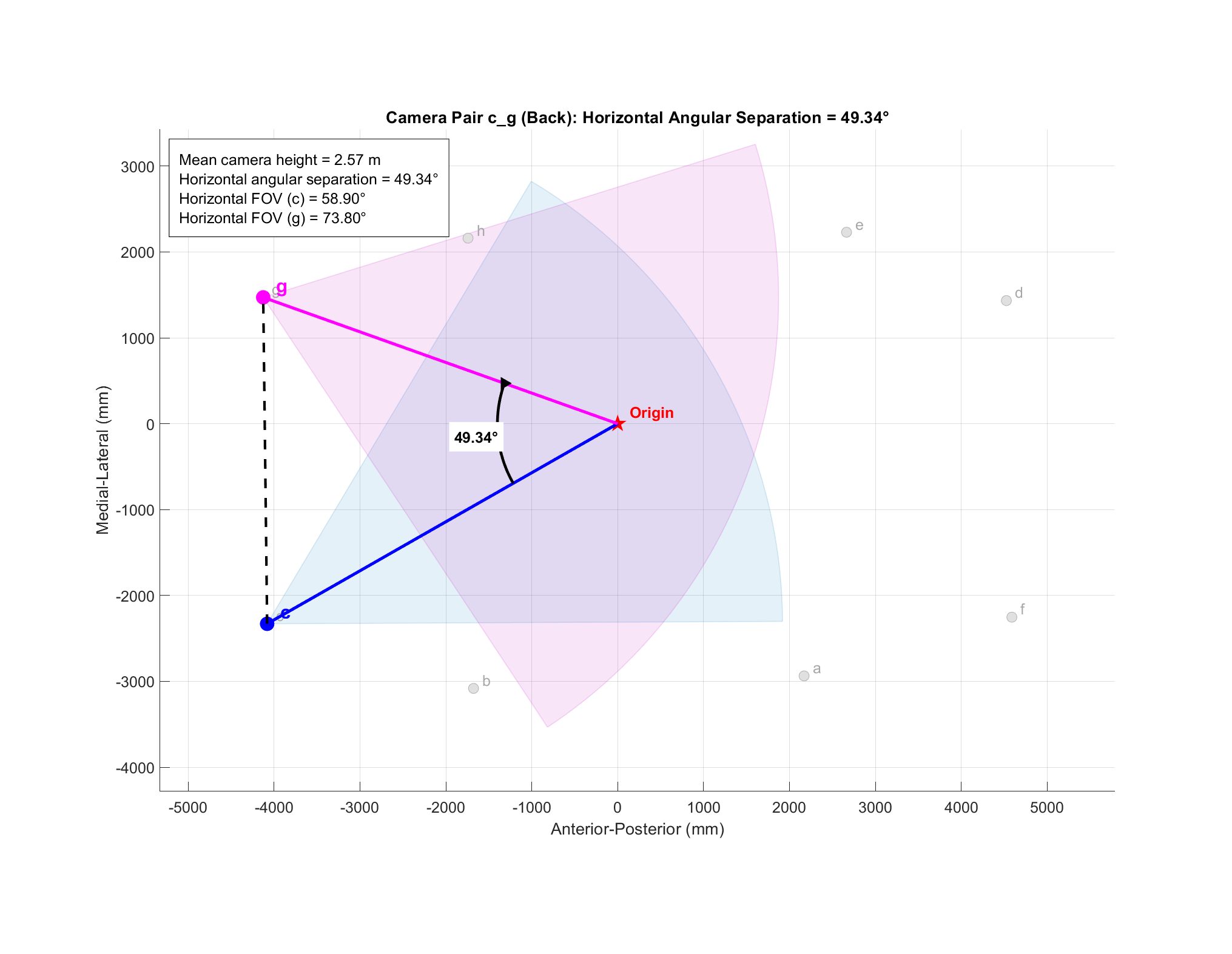


**Figure S7:** Camera pair c_g. Mean camera height was 2.57 m, horizontal angular separation was 49.34°, and horizontal field of view was 58.90° for camera **c** and 73.80° for camera **g**.


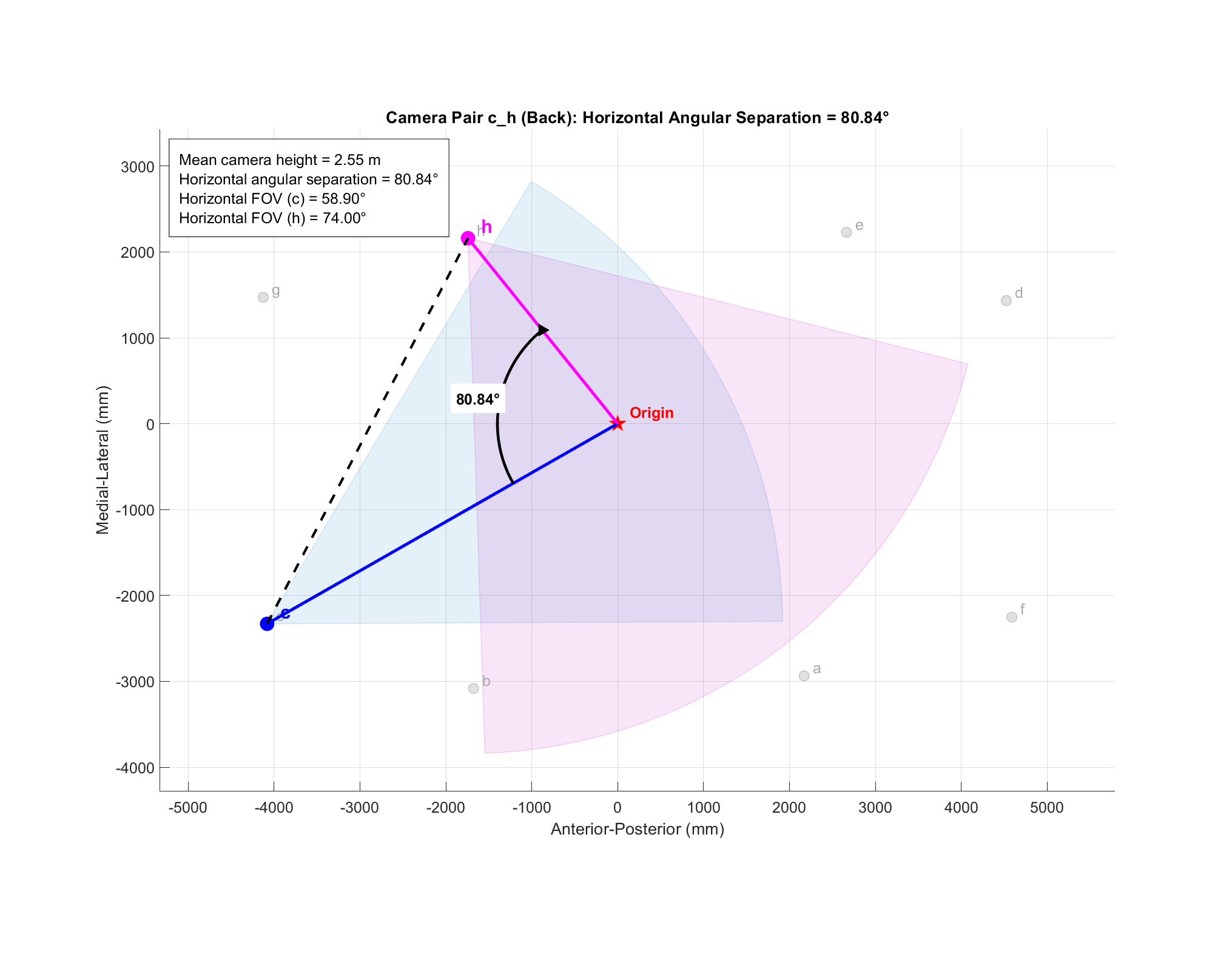


**Figure S8:** Camera pair c_h. Mean camera height was 2.55 m, horizontal angular separation was 80.84°, and horizontal field of view was 58.90° for camera **c** and 74.00° for camera **h**.

**Left subgroup (Figures S9-S12):**


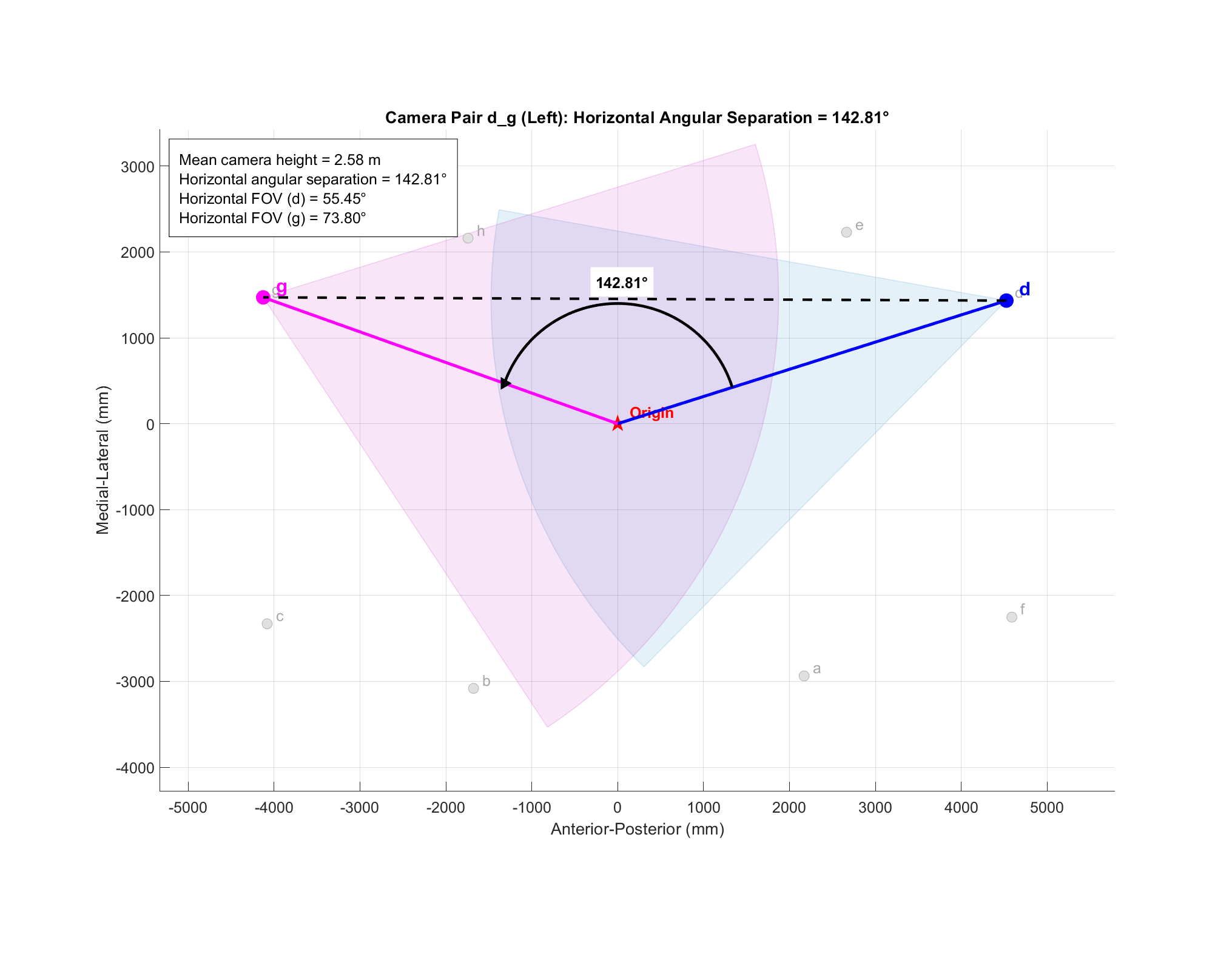


**Figure S9:** Camera pair d_g. Mean camera height was 2.58 m, horizontal angular separation was 142.81°, and horizontal field of view was 55.45° for camera **d** and 73.80° for camera **g**.


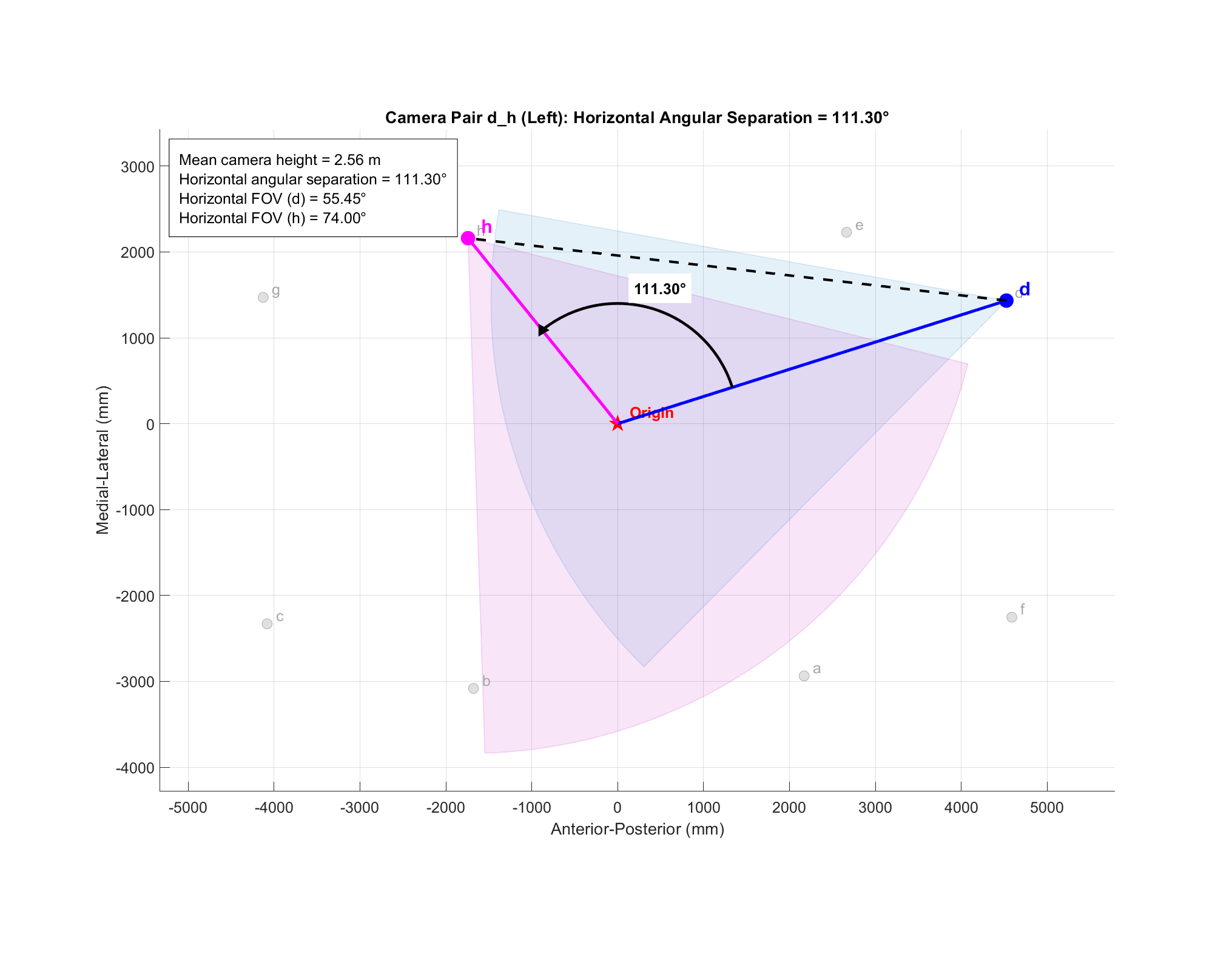


**Figure S10:** Camera pair d_h. Mean camera height was 2.56 m, horizontal angular separation was 111.30°, and horizontal field of view was 55.45° for camera **d** and 74.00° for camera **h**.


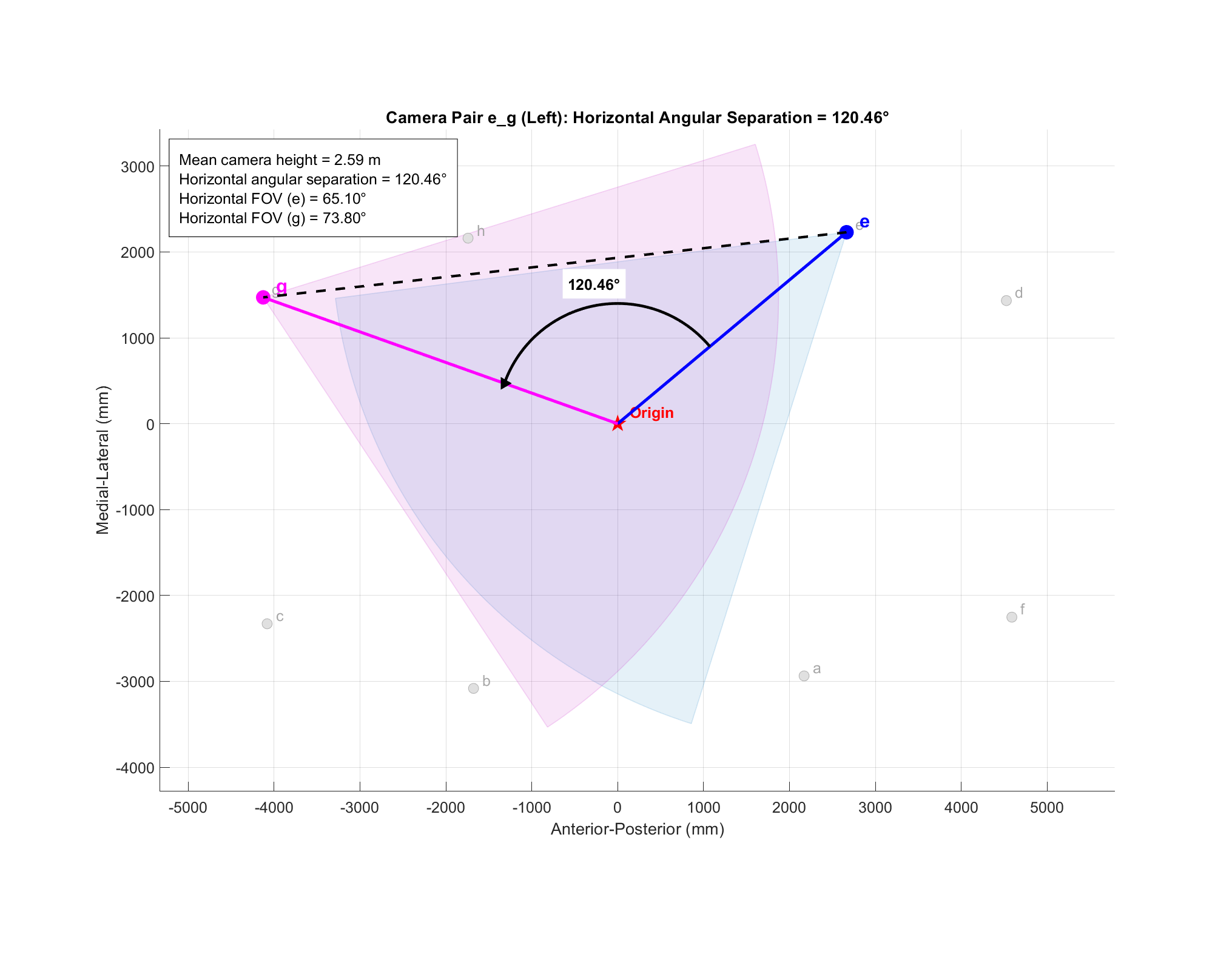


**Figure S11:** Camera pair e_g. Mean camera height was 2.18 m, horizontal angular separation was 71.11°, and horizontal field of view was 72.14° for camera **a** and 55.45° for camera **d**.


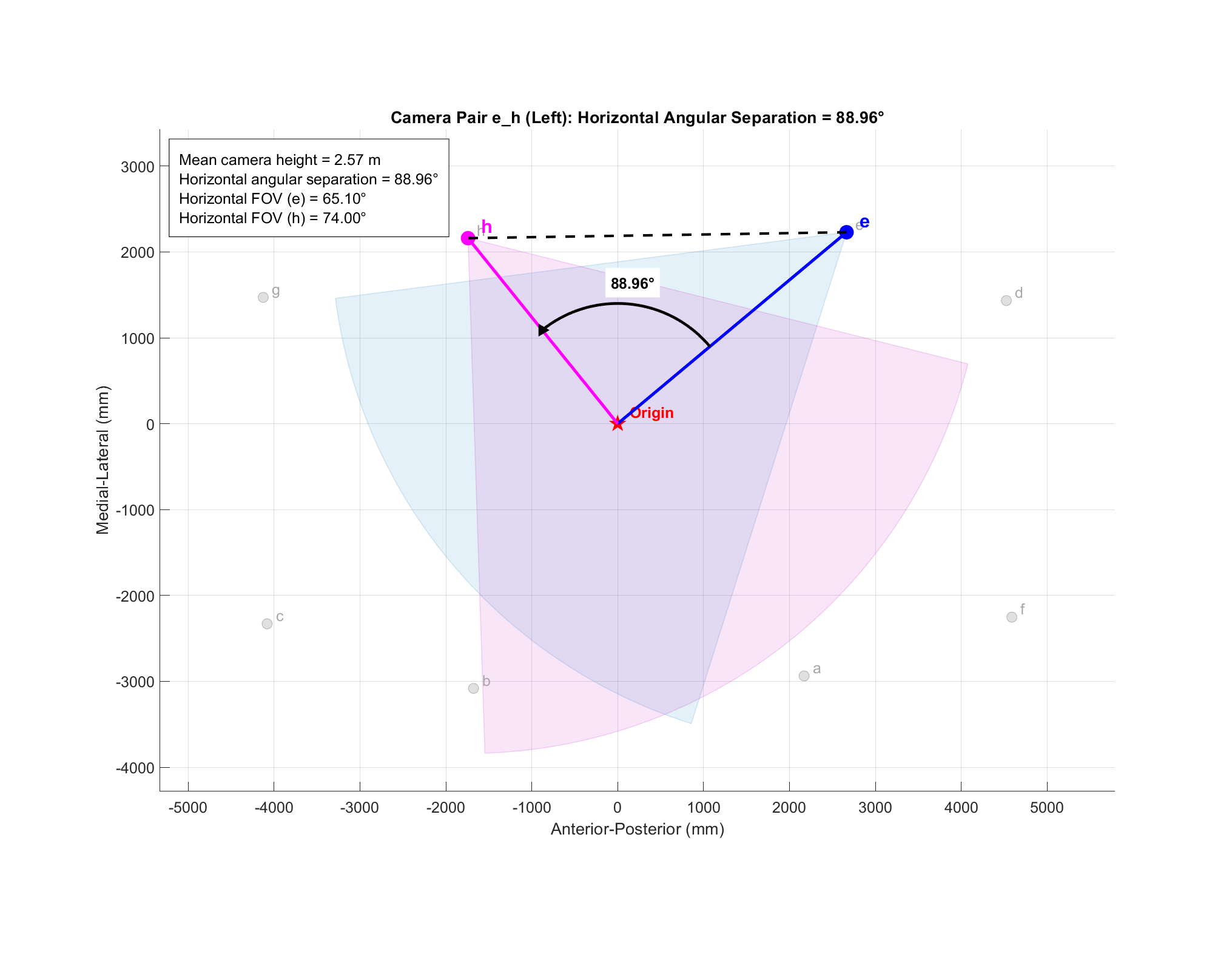


**Figure S12:** Camera pair e_h. Mean camera height was 2.57 m, horizontal angular separation was 88.96°, and horizontal field of view was 65.10° for camera **e** and 74.00° for camera **h**.

**Right subgroup (Figures S13-S16):**


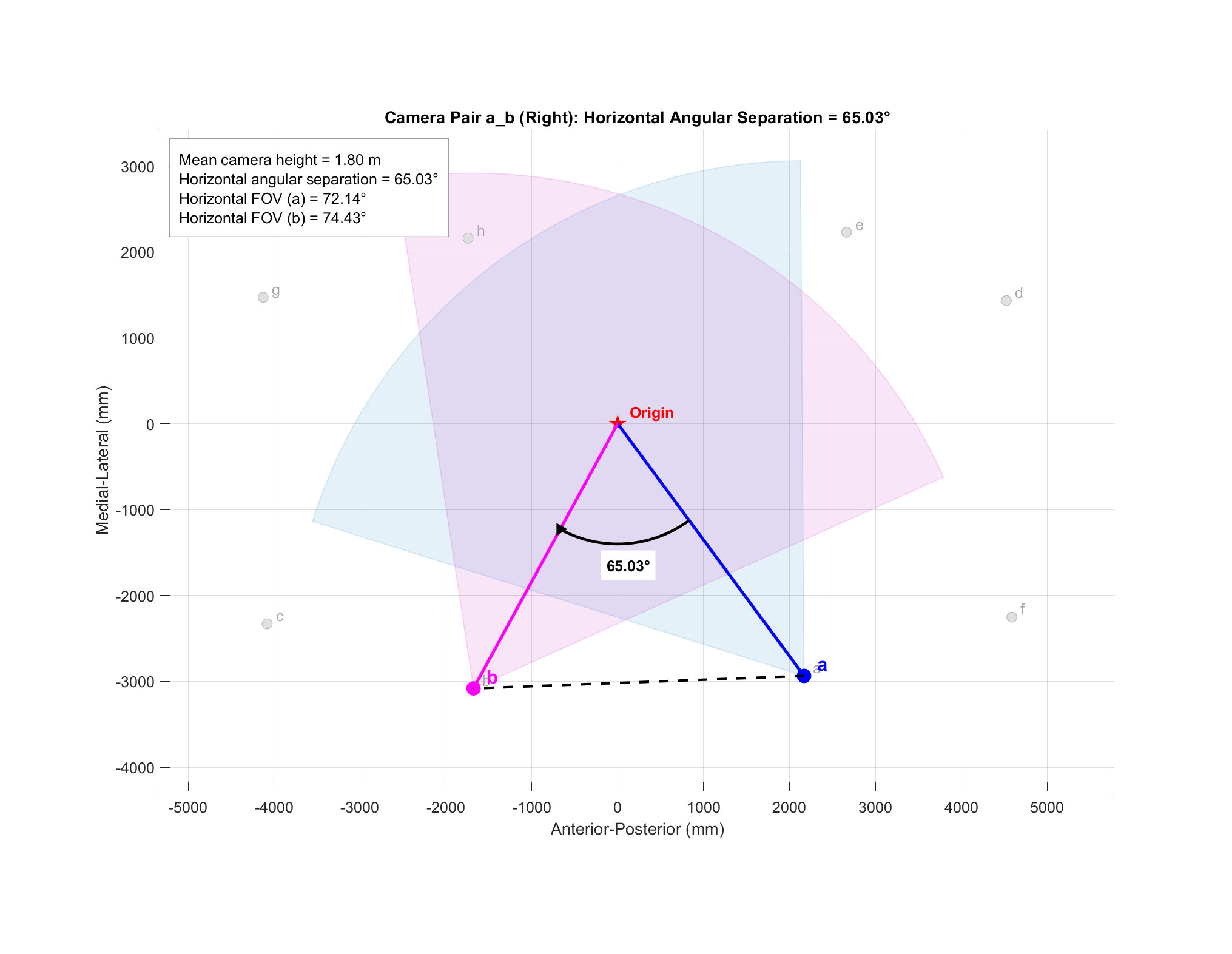


**Figure S13:** Camera pair a_b. Mean camera height was 1.80 m, horizontal angular separation was 65.03°, and horizontal field of view was 72.14° for camera **a** and 74.43° for camera **b**.


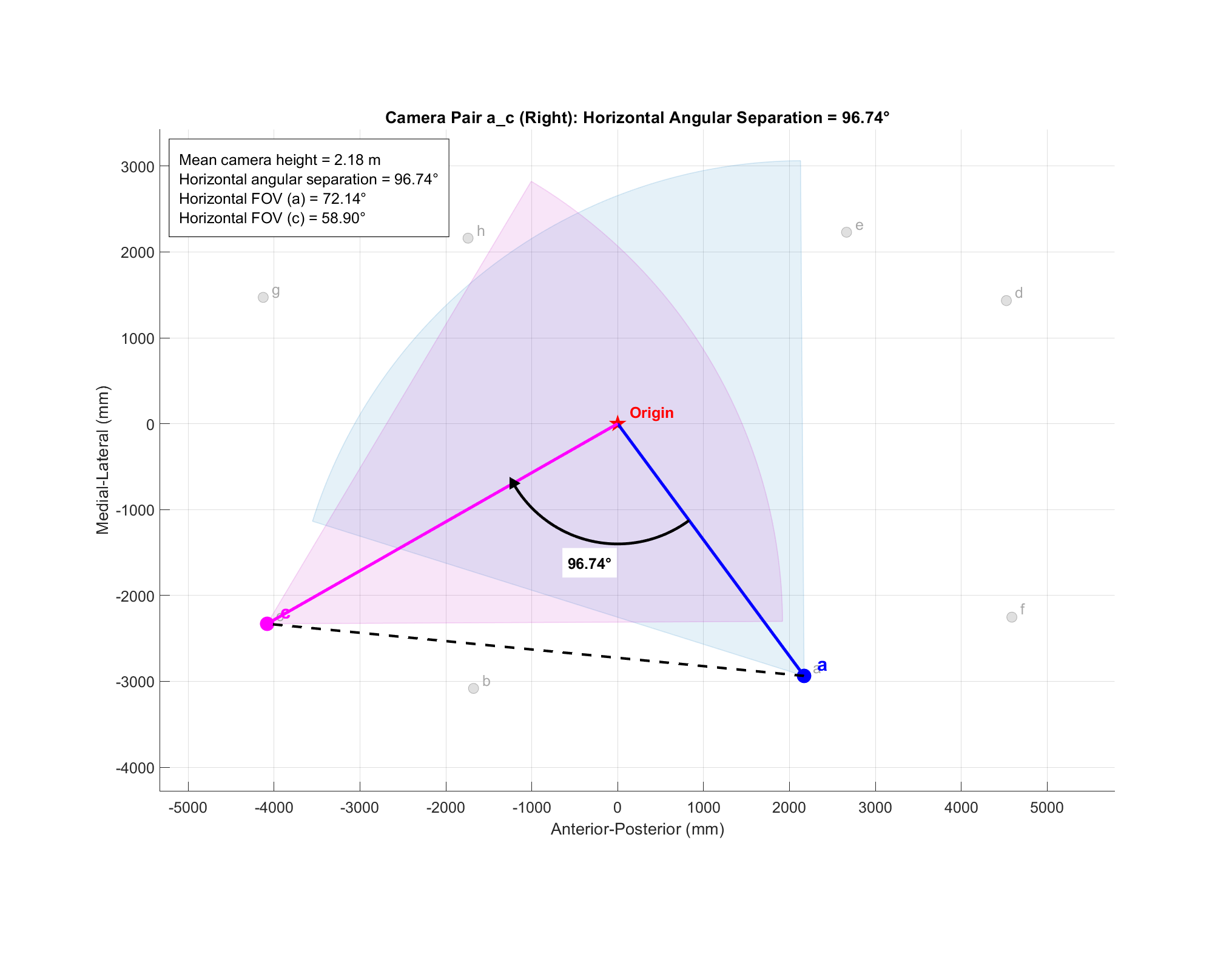


**Figure S14:** Camera pair a_c. Mean camera height was 2.18 m, horizontal angular separation was 96.74°, and horizontal field of view was 72.14° for camera **a** and 58.90° for camera **c**.


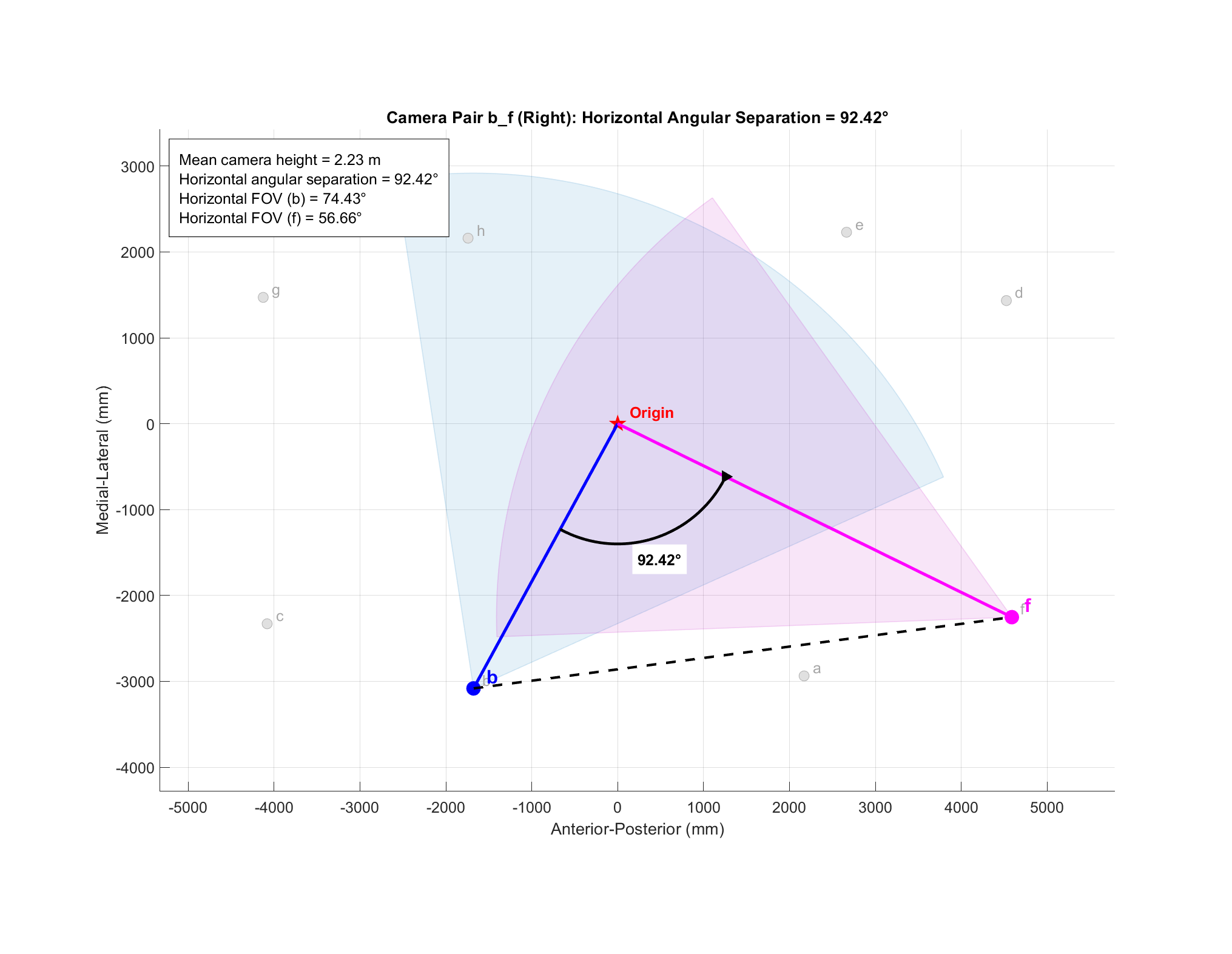


**Figure S15:** Camera pair b_f. Mean camera height was 2.23 m, horizontal angular separation was 92.42°, and horizontal field of view was 74.43° for camera **b** and 56.66° for camera **f**.


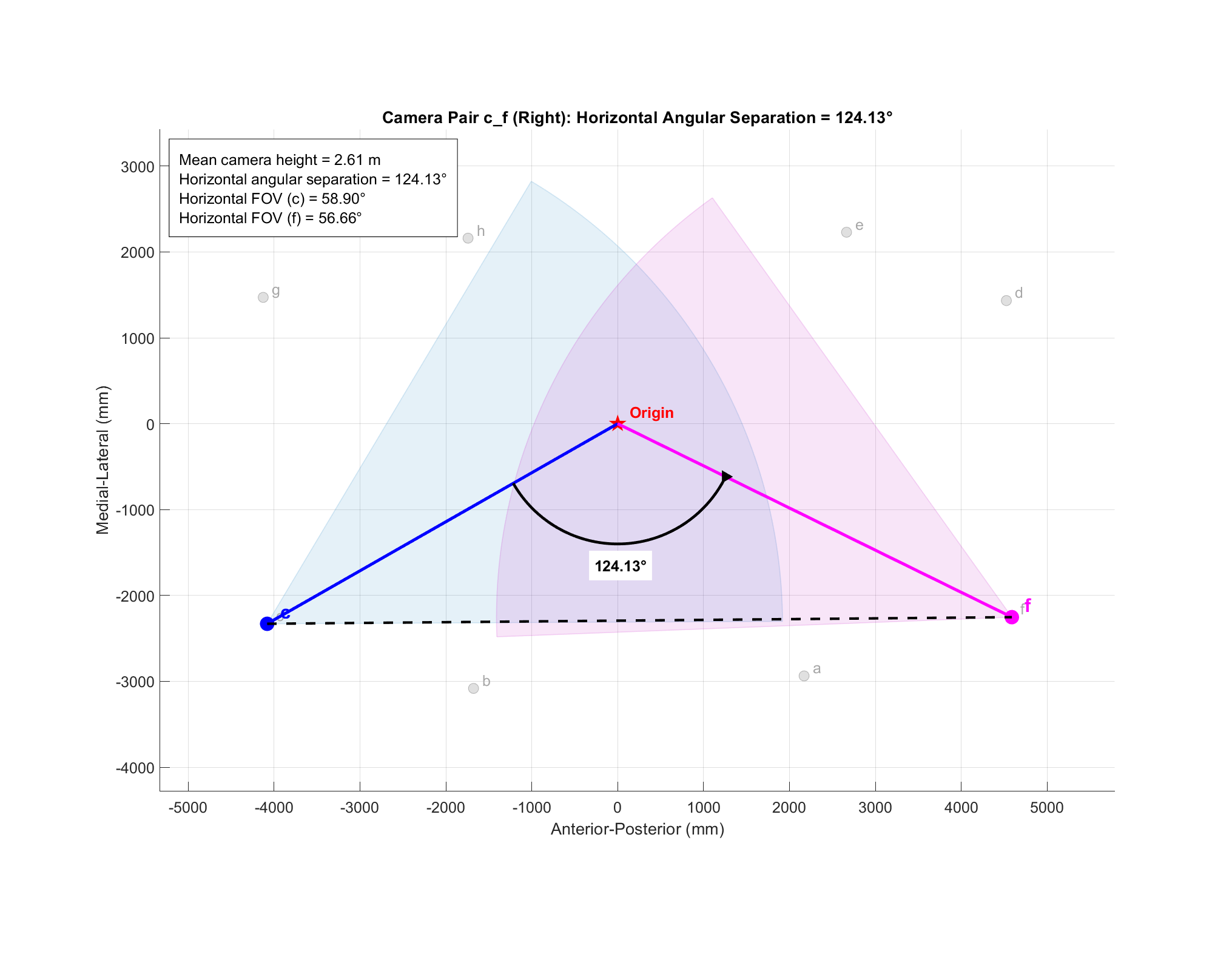


**Figure S16:** Camera pair c_f. Mean camera height was 2.61 m, horizontal angular separation was 124.13°, and horizontal field of view was 58.90° for camera **c** and 56.66° for camera **f**.

**Same quadrant subgroup (Figures S17-S20):**


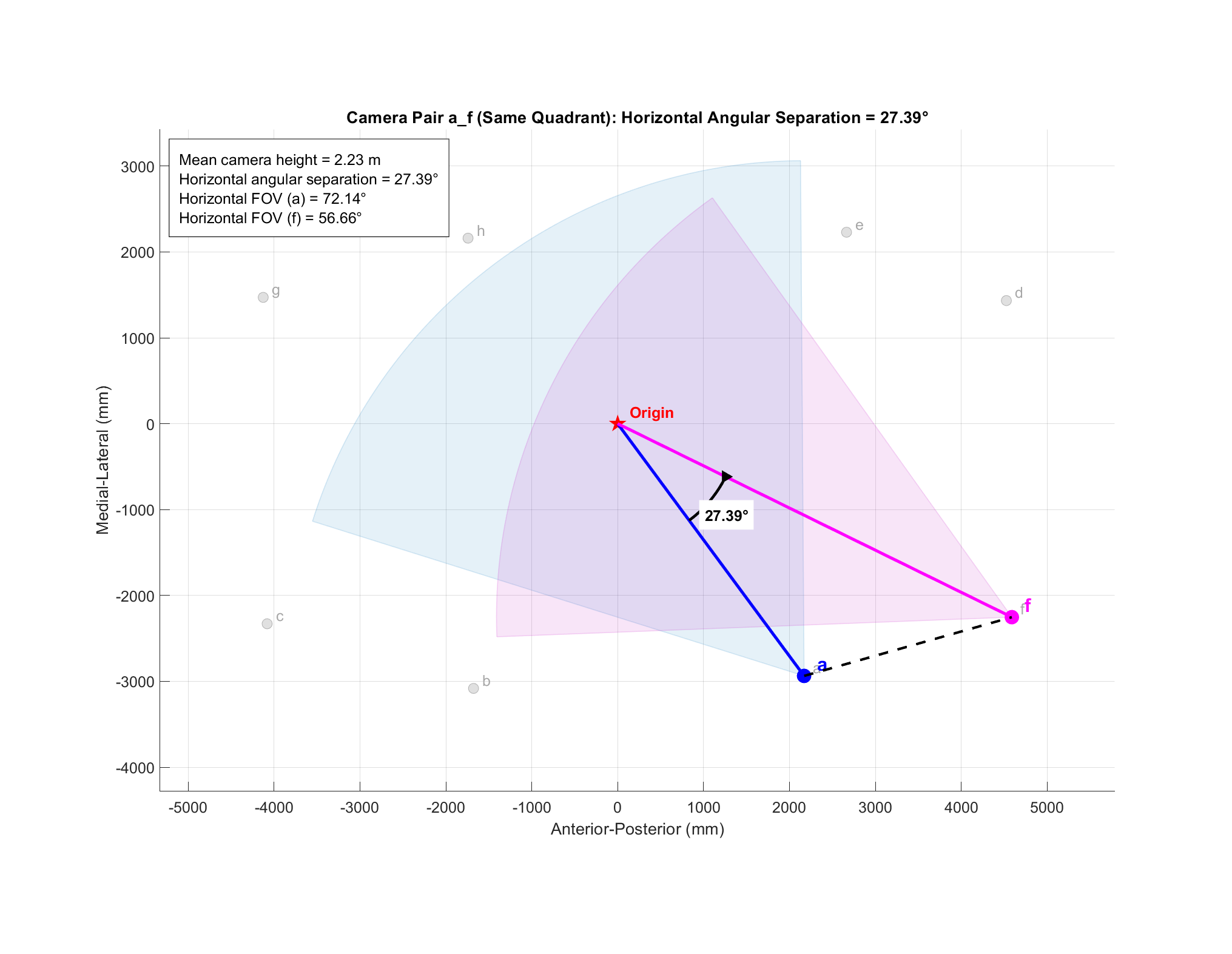


**Figure S17:** Camera pair a_f. Mean camera height was 2.23 m, horizontal angular separation was 27.39°, and horizontal field of view was 72.14° for camera **a** and 56.66° for camera **f**.


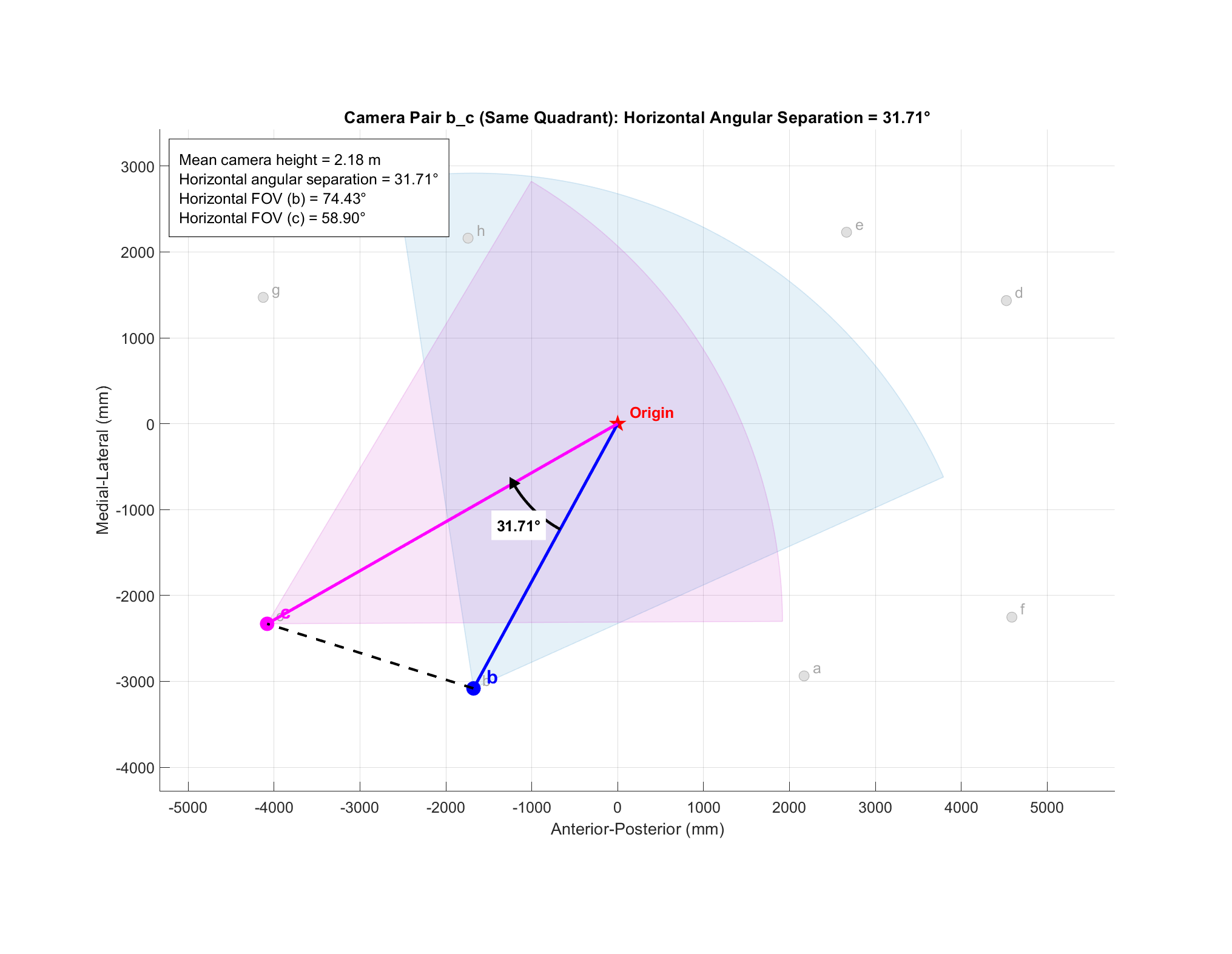


**Figure S18:** Camera pair b_c. Mean camera height was 2.18 m, horizontal angular separation was 31.71°, and horizontal field of view was 74.43° for camera **b** and 58.90° for camera **c**.


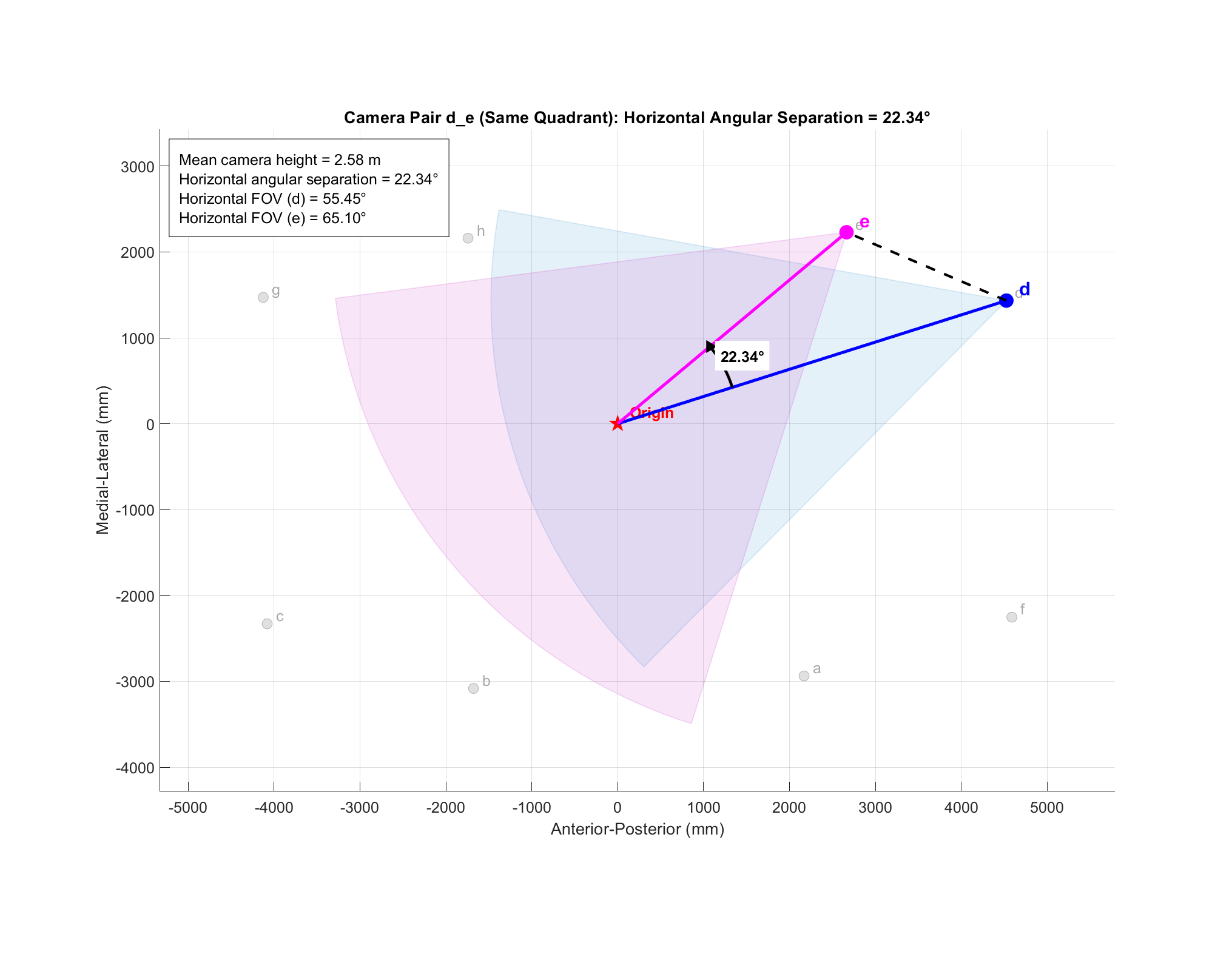


**Figure S19:** Camera pair d_e. Mean camera height was 2.58 m, horizontal angular separation was 22.34°, and horizontal field of view was 55.45° for camera **d** and 65.10° for camera **e**.


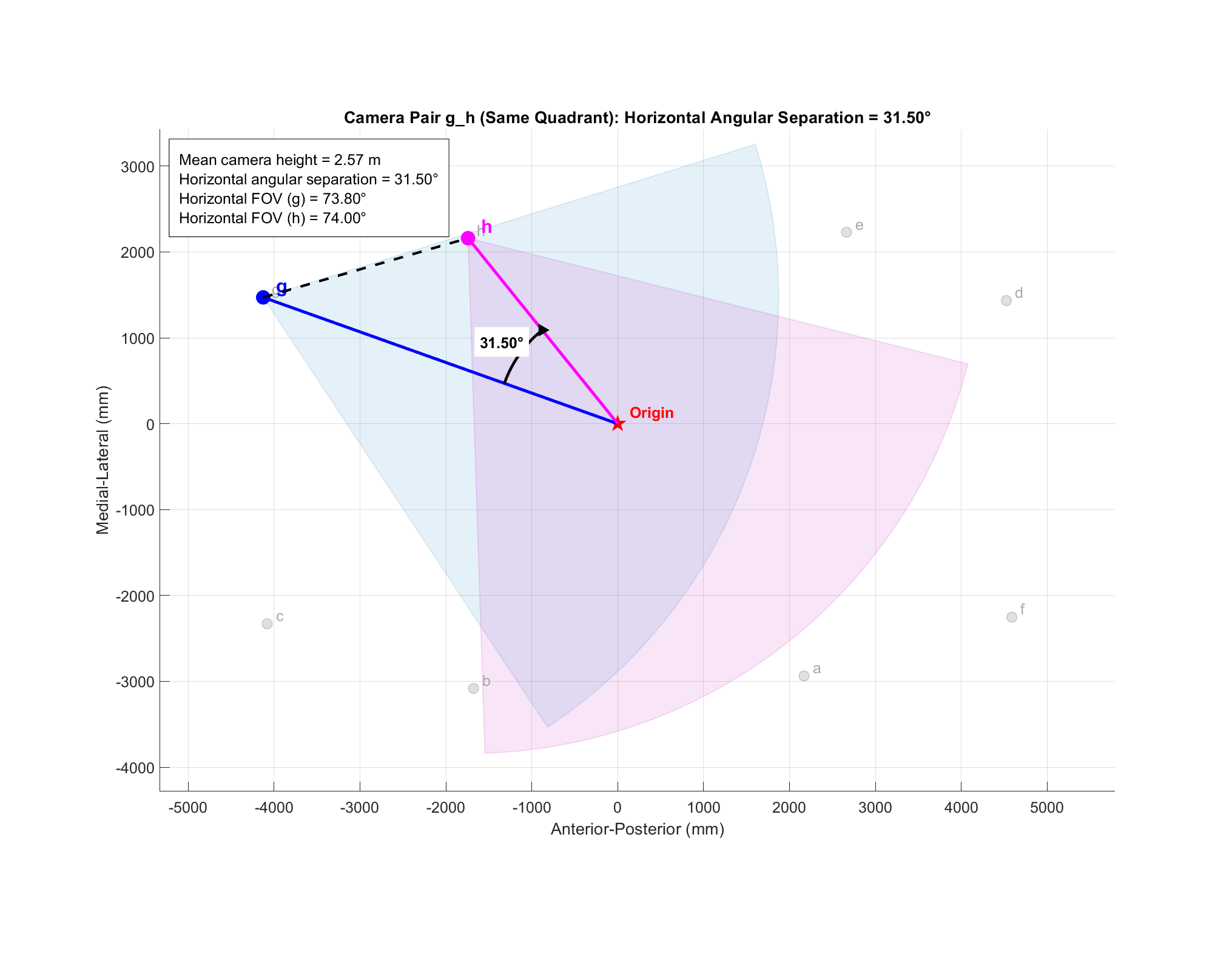


**Figure S20:** Camera pair g_h. Mean camera height was 2.57 m, horizontal angular separation was 31.50°, and horizontal field of view was 73.80° for camera **g** and 74.00° for camera **h**.

**Diagonal subgroup (Figures S21-S28):**


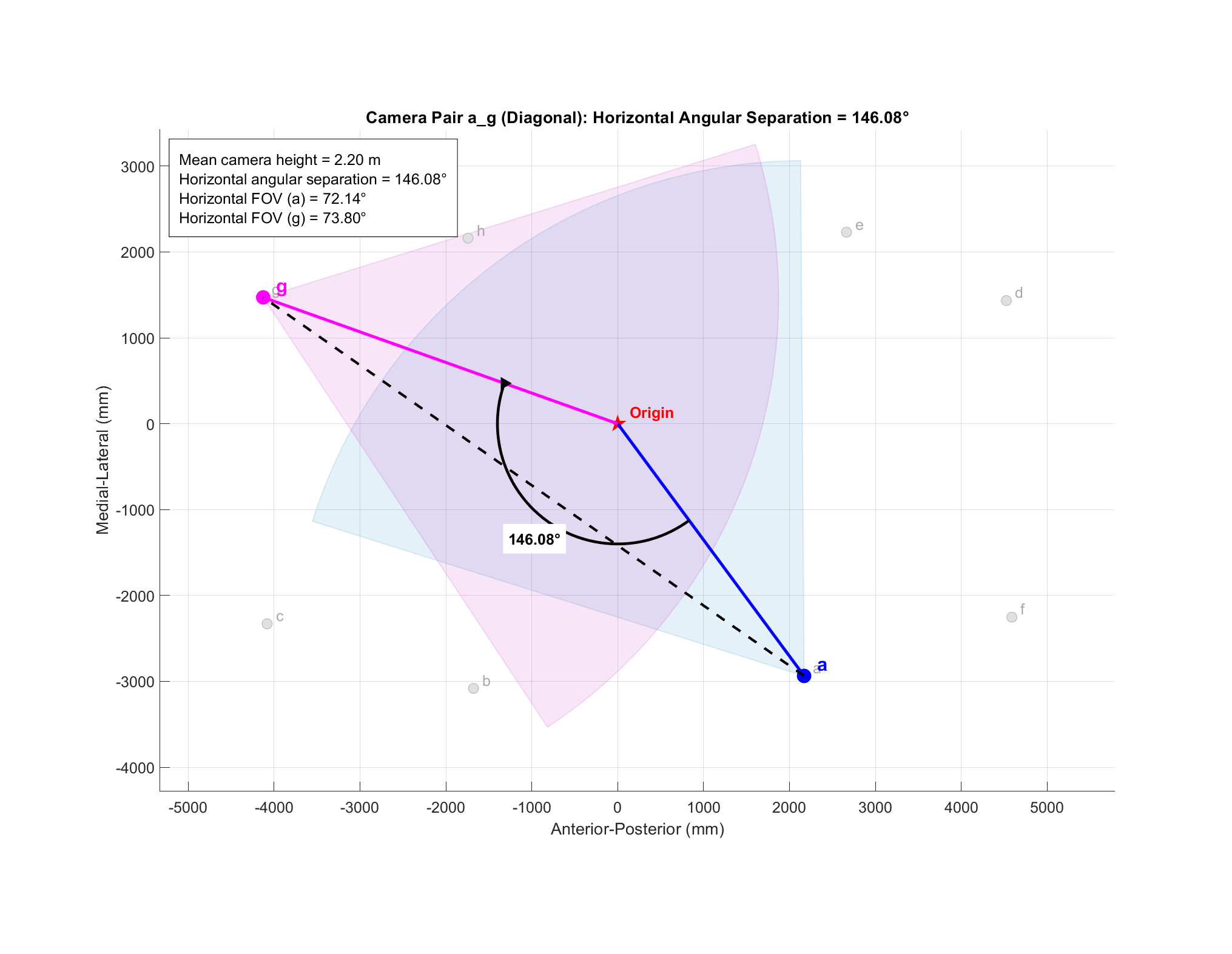


**Figure S21:** Camera pair a_g. Mean camera height was 2.20 m, horizontal angular separation was 146.08°, and horizontal field of view was 72.14° for camera **a** and 73.80° for camera **g**.


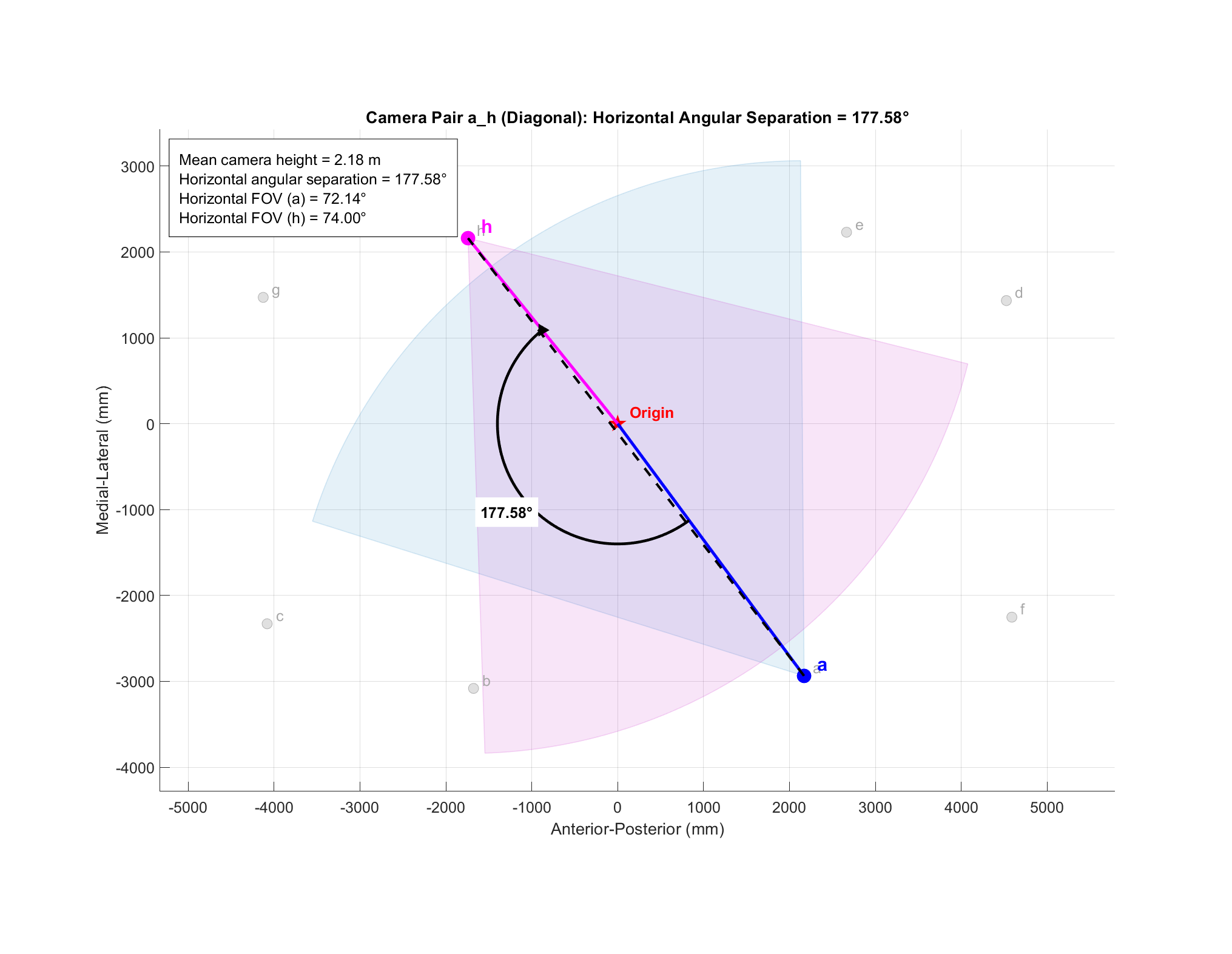


**Figure S22:** Camera pair a_h. Mean camera height was 2.18 m, horizontal angular separation was 177.58°, and horizontal field of view was 72.14° for camera **a** and 74.00° for camera **h**.


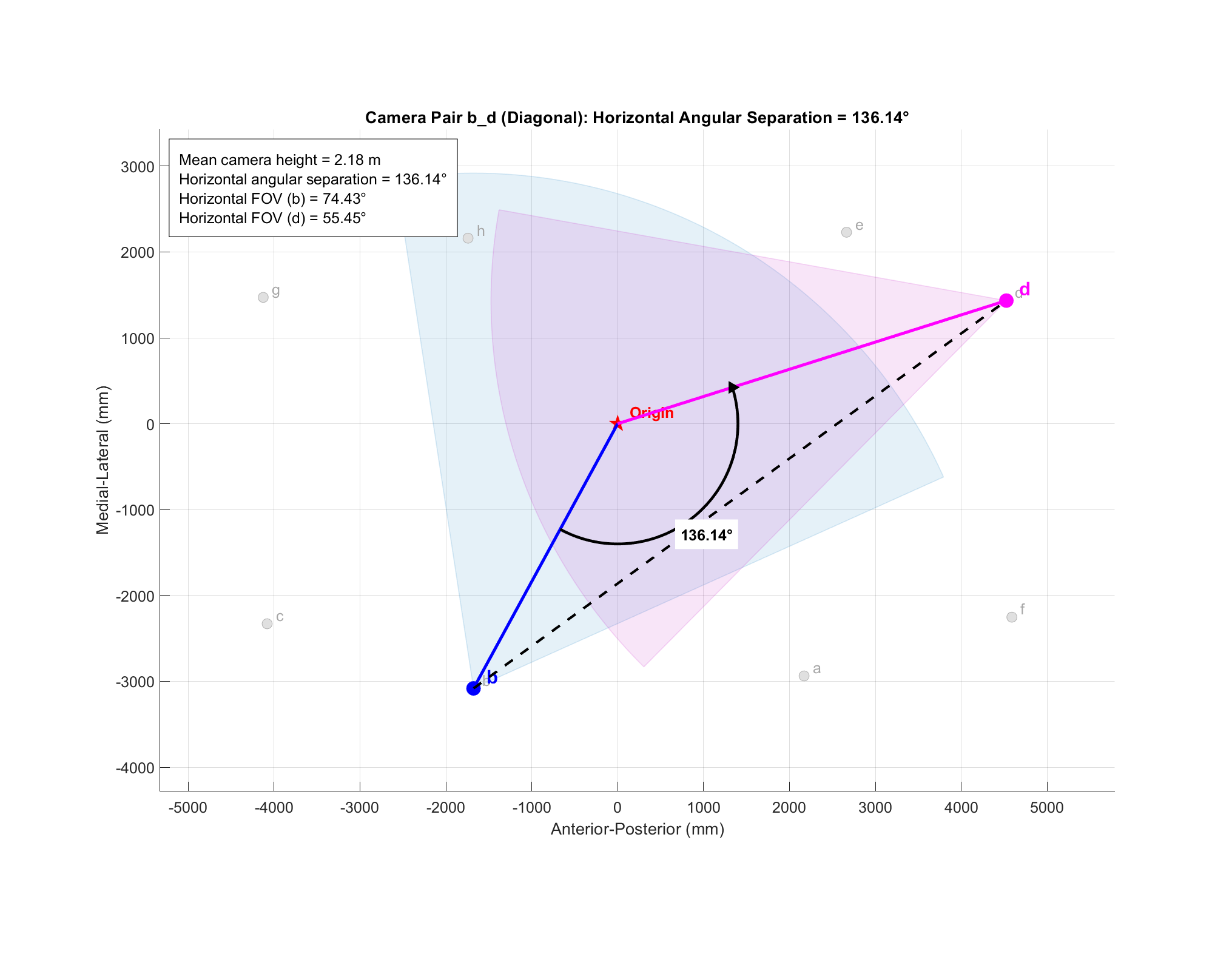


**Figure S23:** Camera pair b_d. Mean camera height was 2.18 m, horizontal angular separation was 136.14°, and horizontal field of view was 74.43° for camera **b** and 55.45° for camera **d**.


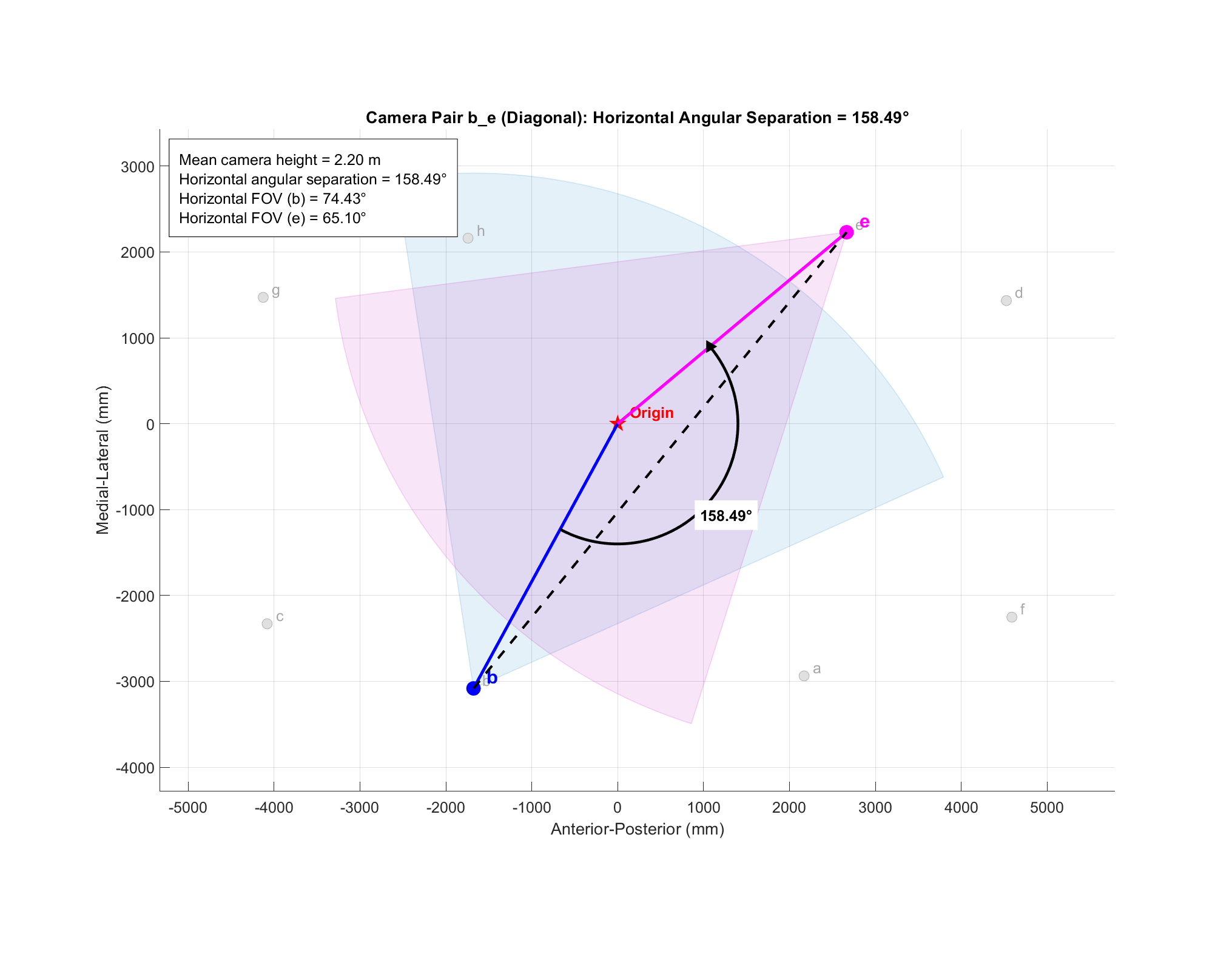


**Figure S24:** Camera pair b_e. Mean camera height was 2.20 m, horizontal angular separation was 158.49°, and horizontal field of view was 74.43° for camera **b** and 65.10° for camera **e**.


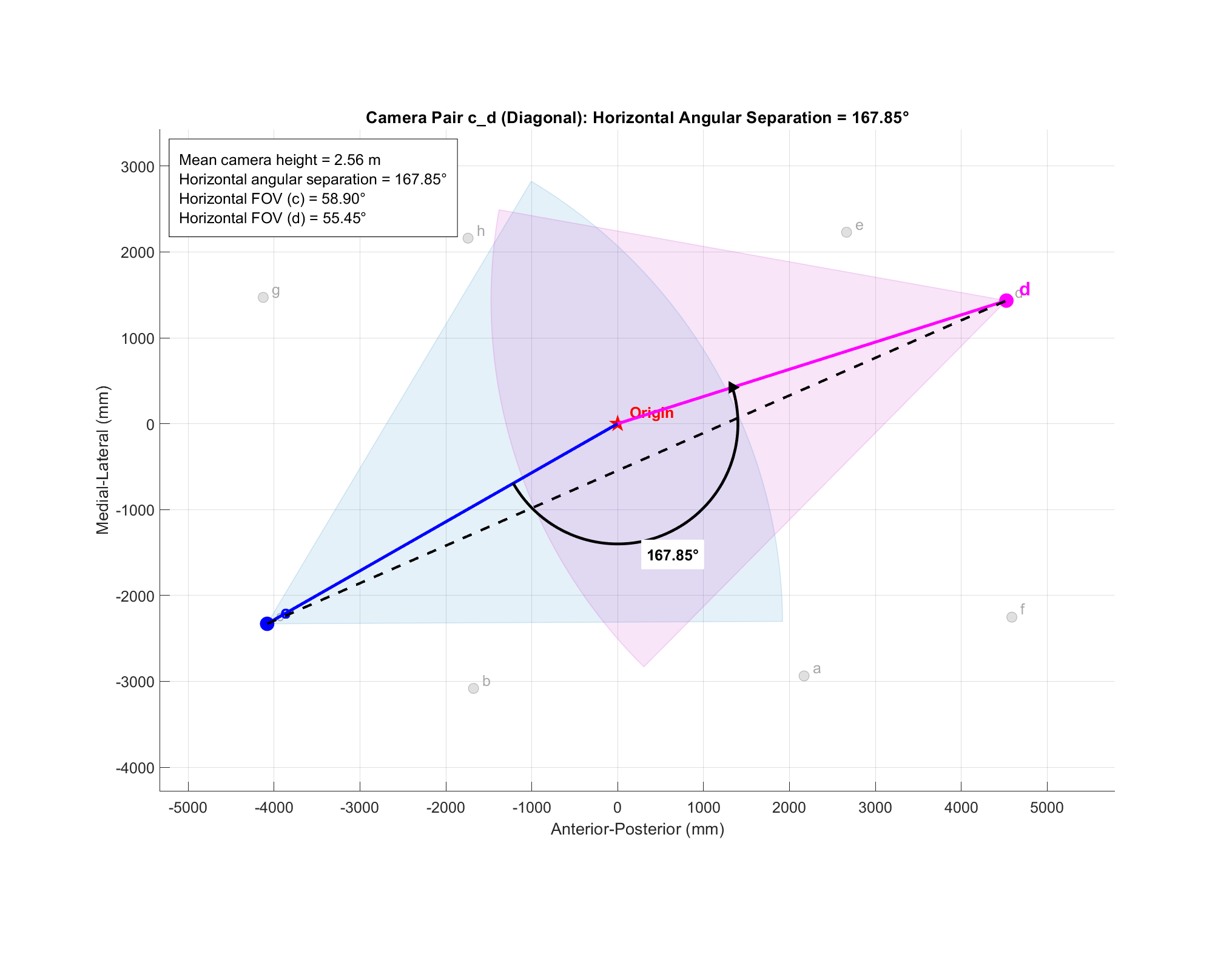


**Figure S25:** Camera pair c_d. Mean camera height was 2.56 m, horizontal angular separation was 167.85°, and horizontal field of view was 58.90° for camera **c** and 55.45° for camera **d**.


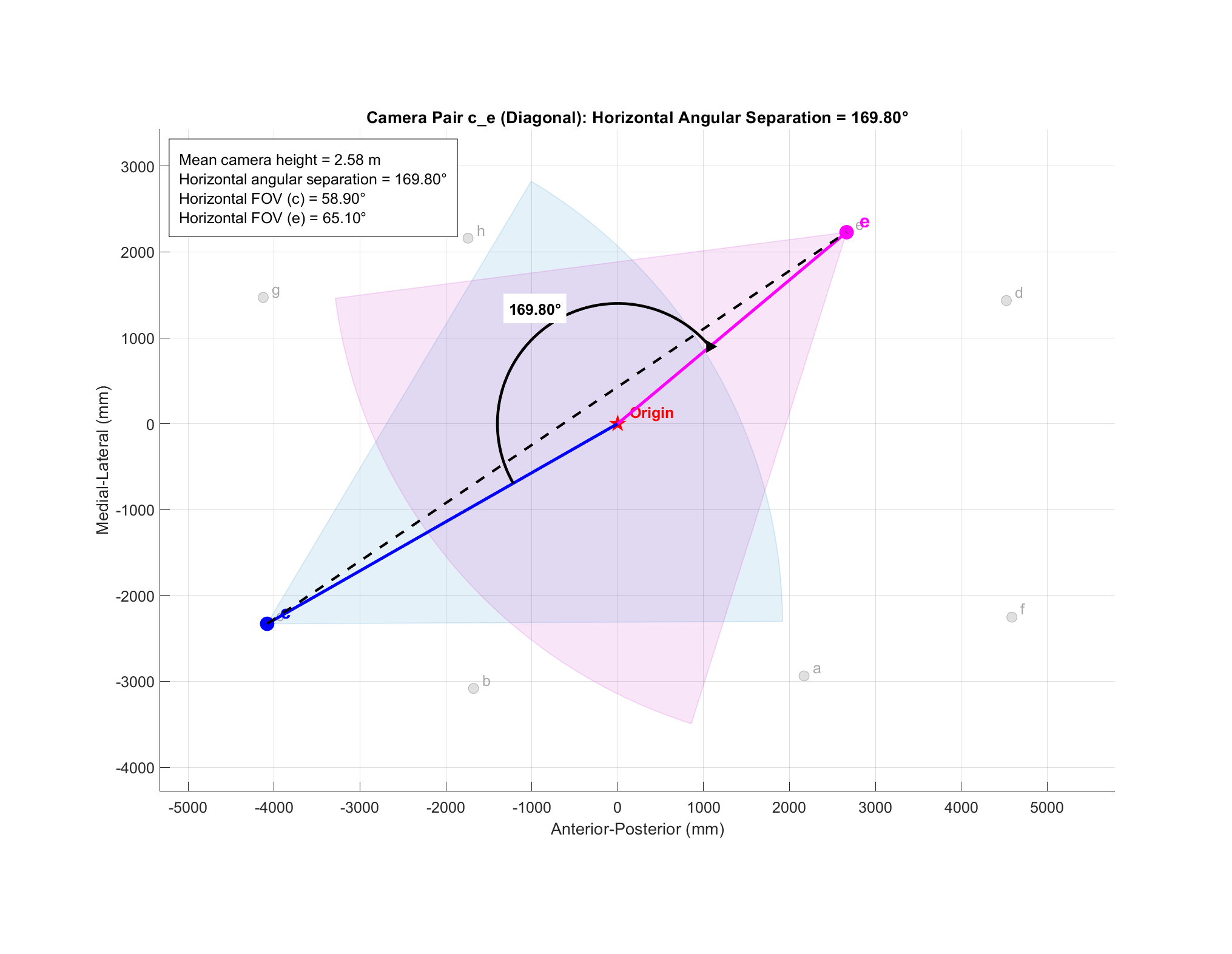


**Figure S26:** Camera pair c_e. Mean camera height was 2.58 m, horizontal angular separation was 169.80°, and horizontal field of view was 58.90° for camera **c** and 65.10° for camera **e**.


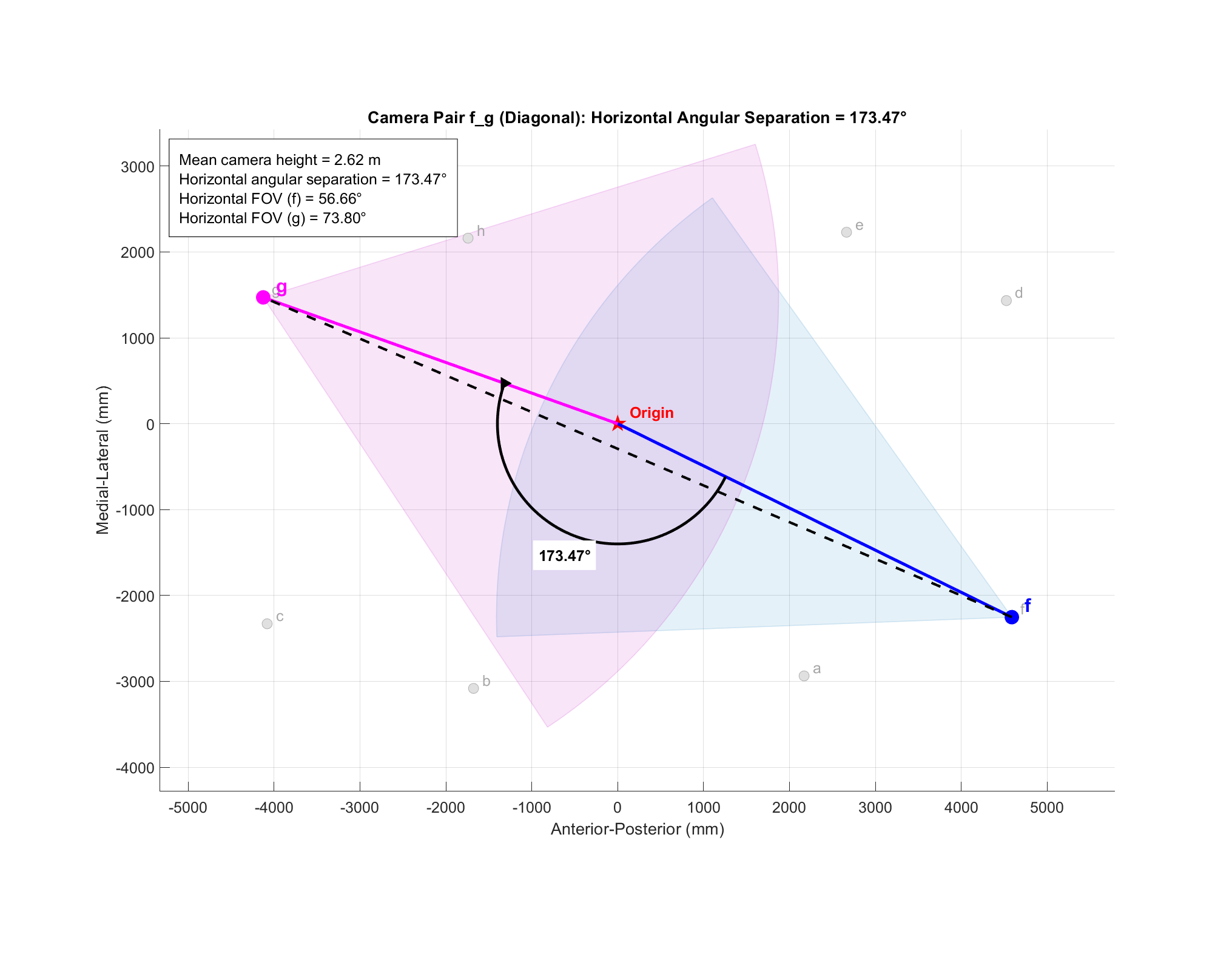


**Figure S27:** Camera pair f_g. Mean camera height was 2.62 m, horizontal angular separation was 173.47°, and horizontal field of view was 56.66° for camera **f** and 73.80° for camera **g**.


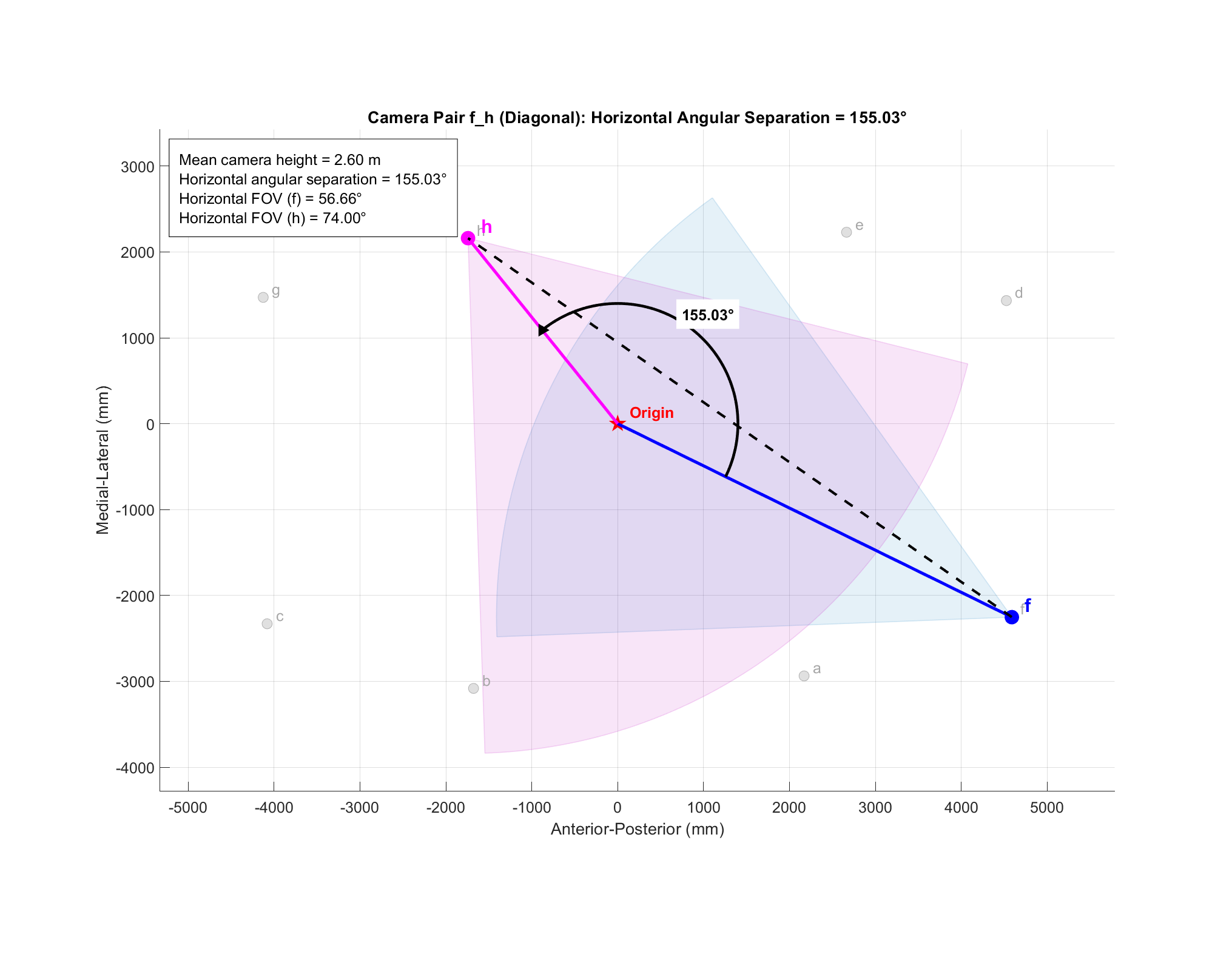


**Figure S28:** Camera pair f_h. Mean camera height was 2.60 m, horizontal angular separation was 155.03°, and horizontal field of view was 56.66° for camera **f** and 74.00° for camera **h**.
